# Hemeroby reveals the dynamics of vegetation cover following the destruction of the Kakhovka Reservoir

**DOI:** 10.7717/peerj.19607

**Published:** 2025-06-25

**Authors:** Olena Lisovets, Serhiy Podorozhniy, Hanna Tutova, Karina Molozhon, Olha Kunakh, Olexander Zhukov

**Affiliations:** 1 Department of Biodiversity and Ecology, Oles Honchar Dnipro National University, Dnipro, Dnipropetrovsk Region, Ukraine; 2 Department of Botany, Ecology and Gardening, Bogdan Khmelnitsky Melitopol State Pedagogical University, Zaporizhzhia City, Zaporizhzhia Region, Ukraine

**Keywords:** Hemeroby, Naturalness, Succession, Humidity, Floodplain ecosystems, Anthropogenic impact

## Abstract

Floodplain ecosystems play a crucial role in maintaining ecological balance by regulating hydrological regimes, conserving biodiversity, and providing essential ecosystem services. The destruction of the Kakhovka Reservoir in June 2023 resulted in a large-scale environmental disaster that profoundly affected both aquatic and terrestrial ecosystems in the Lower Dnipro region. This study was conducted in the floodplain ecosystems of Khortytsia Island (Ukraine) to assess vegetation changes in response to altered hydrological conditions. Phytosociological mapping of the vegetation cover was performed, and the concept of hemeroby was employed to evaluate the level of anthropogenic transformation of plant communities. Phytoindication scales were utilized to characterize environmental conditions, including humidity, light, and soil properties. Following the destruction of the reservoir, significant changes in the water regime led to the degradation of floodplain water bodies, the expansion of sandy open areas, and the formation of new ecotopes. Twelve distinct plant community associations were identified, each differing in ecological preferences and sensitivity to anthropogenic disturbances. Principal component analysis revealed that most of the variability in plant community composition is explained by gradients in humidity, trophic status, and light availability, all of which are closely associated with hemeroby and naturalness indices. These findings underscore the dominant influence of recent anthropogenic drivers on vegetation structure. A decline in water availability following the reservoir’s destruction emerged as a primary driver of vegetation dynamics in the affected areas. Coastal zones experienced the most pronounced changes, where newly exposed sandy substrates were rapidly colonized by xerophytic and ruderal species, forming highly hemerobic communities. In contrast, more stable conditions in the central part of the island supported the persistence of relatively undisturbed plant assemblages. Increased light levels and elevated soil nutrient content in open areas were also found to promote the spread of species with high hemeroby, reinforcing the connection between altered abiotic conditions and anthropogenic transformation. This study demonstrates that the collapse of the Kakhovka Reservoir triggered rapid shifts in floodplain vegetation, with moisture regime, illumination, and nutrient availability acting as key environmental filters. The observed correspondence between these factors and the level of hemeroby confirms the utility of hemeroby and naturalness indices as effective tools for monitoring post-catastrophic ecological changes and informing future strategies for ecosystem restoration.

## Introduction

Floodplain ecosystems provide essential ecological functions, including the regulation of water flows, enhancement of water quality, promotion of biodiversity, and support for various ecosystem processes (Petsch et al., 2023). These serve as natural buffers, mitigating river flows and retaining floodwaters, which help prevent flooding (Kiedrzyńska, Kiedrzyński & Zalewski, 2015). Due to their capacity to store and retain water, floodplains contribute to groundwater recharge and reduce the risk of droughts (Ward et al., 2020). These ecosystems are crucial in water filtration (Jakubínský et al., 2021). Floodplains capture and hold excess nutrients, toxic substances, and sediment, blocking their flow into rivers and lakes (Cocco et al., 2020). These areas are unique biodiversity hotspots (Schindler et al., 2016), providing habitats for many plant and animal species, especially those dependent on seasonal changes in water levels (Havrdová, Douda & Doudová, 2023). Floodplains significantly influence the carbon cycle by storing organic matter and aiding in reducing greenhouse gas emissions (Sutfin, Wohl & Dwire, 2016). These environments are essential for conserving fishery resources, as various fish species rely on floodplains for spawning and developing their young (Stoffers et al., 2022). Human societies derive substantial socio-economic benefits from floodplain ecosystems, including water supply, recreational opportunities, and agricultural support (Basak et al., 2021). The degradation of these ecosystems due to drainage, development, and pollution is leading to the loss of their critical functions (Morrison et al., 2023). The conservation and restoration of floodplains are essential for sustainable development and ecological balance (Funk et al., 2019).

The construction of dams and the regulation of river flows significantly negatively impact the biodiversity of floodplain ecosystems, disrupting their natural dynamics and altering the habitats of numerous species (Wu et al., 2019). Dams modify the hydrological regime of rivers, reducing or eliminating seasonal floods, which are crucial for maintaining floodplain ecosystems (Ely et al., 2020). This alteration results in the desiccation of floodplain meadows, marshes, and lakes, diminishing the habitat for waterfowl, amphibians, fish, and invertebrates (Wenskus et al., 2025). Many fish species, including those that rely on floodwaters for spawning, lose their breeding grounds, leading to population declines or even local extinctions (Zymaroieva et al., 2024). The changes in runoff regulation alter sedimentation processes, affecting the fertility of floodplain soils and the composition of the flora (Yakovenko et al., 2023). The variability in the dynamics of nutrients and organic matter leads to food supply degradation for many animal species inhabiting floodplains (Bastviken et al., 2023). The creation of artificial reservoirs modifies the structure of the aquatic ecosystem: the flow slows down, pollution accumulates, and oxygen levels are depleted, adversely impacting both aquatic flora and fauna (Guo et al., 2021). Dams create physical barriers that impede the migration of aquatic organisms, disrupting ecological connections between river sections and resulting in populations’ genetic isolation (Chen et al., 2023). Migratory fish, such as sturgeons, cannot move upstream to spawn, contributing to a decline in their populations (Auer, 1996). The alterations in water temperature caused by the accumulation of large volumes in reservoirs modify the living conditions for many aquatic organisms, leading to the displacement of native species and the proliferation of invasive ones (Bondarev et al., 2023). Building dams to control river flows reduces floodplain ecosystem services, weakened resilience of biocenoses, deterioration of natural landscapes, and a decline in overall environmental health biodiversity (Pelletier et al., 2020). This negatively impacts natural ecosystems and the socio-economic status of local societies that rely on river resources (Pichura, Potravka & Boiko, 2025). In turn, dam removal has cost and environmental and socio-economic implications. These consequences can have both positive and negative impacts on the economy and nature of the region (Perera & North, 2021).

The Kakhovka hydroelectric dam explosion in June 2023 resulted in a large-scale environmental disaster with long-term consequences for water resources, biodiversity, and floodplain ecosystems (Yutilova et al., 2025; Shumilova et al., 2025). The sudden, uncontrolled release of water led to the flooding of extensive areas downstream along the Dnipro River, causing bank erosion and the destruction of floodplain forests, swamps, and meadows that served as habitats for numerous species of flora and fauna (Chernogor et al., 2024). After the water receded, vast areas of dried and saline land emerged, leading to the degradation of natural landscapes (Vyshnevskyi & Shevchuk, 2024). Mass mortality among aquatic fauna represented one of the most severe environmental losses, as millions of fish, molluscs, and other aquatic organisms perished due to the sudden drainage of the reservoir, to which they could not adapt (Dovhanenko et al., 2024). The disruption of the hydrological regime has negatively impacted the migration of waterfowl that relied on the reservoir for resting and feeding (Yutilova et al., 2025). Water pollution became another critical challenge after the dam was blown up (Marenkov et al., 2025). Substantial amounts of silt deposits, oil products, pesticides, heavy metals, and domestic wastewater have been washed into the lower reaches of the Dnipro River and subsequently into the Black Sea (Kvach et al., 2025). Recent modeling and satellite monitoring studies indicate that the former Kakhovka Reservoir continues to be a long-term source of environmental contamination due to the release of sediments rich in toxic substances. Prior to the dam’s collapse, large volumes of hazardous materials, including heavy metals and nitrogen and phosphorus compounds, had accumulated in the reservoir. It is estimated that the total volume of bottom sediments reaches between 1.3 and 1.7 km^3^, containing approximately 83,000 tons of toxic metals, of which less than 1% was released during the dam failure. Seasonal flooding, wind erosion, and biological processes may further mobilize these sediments, posing an ongoing threat to water quality, biodiversity, and human health in downstream regions (Shumilova et al., 2025). This has also contributed to toxic algal blooms and the mortality of aquatic organisms (Pichura, Potravka & Dent, 2025). Many riparian areas have been deprived of access to fresh water, as the Kakhovka Reservoir served as a vital water supply for millions of people (Pichura et al., 2024c). Its destruction has led to regional desiccation and a marked deterioration in drinking water quality. The destruction of the reservoir has disrupted the irrigation system, rendering vast areas of agricultural land unusable (Pichura et al., 2024b). The salinisation of the soil due to a drop in the water table has further exacerbated the situation (Pichura et al., 2024a). The destruction of ecosystems and the loss of habitats for rare and endangered species have severely diminished the region’s biodiversity. The disruption of natural ecological connections has made the restoration of these areas extremely challenging. The impact of the disaster has even extended to the Black Sea, where large quantities of contaminated water have entered, altering its ecosystem and posing a threat to marine life (Xu, Barbot & Wang, 2024). The consequences of the Kakhovka HPP destruction are profoundly serious and will require decades for recovery (Symochko et al., 2024). The loss of reservoirs, floodplain ecosystems, and biodiversity is irreversible in many respects, significantly compromising the region’s ecological balance.

The destruction of the Kakhovka Reservoir and subsequent alterations to the hydrological regime have resulted in the exposure of previously submerged areas and the transformation of aquatic habitats into terrestrial ones, leading to the formation of new, temporarily or permanently dry ecotopes (Didukh et al., 2024b). The dynamics of plant communities in these areas remain poorly understood and present several unresolved issues. One of the primary challenges is the succession processes occurring on the bare substrate of the former reservoir, where unstable plant communities may develop due to abrupt environmental changes (Didukh et al., 2024a). The mechanisms underlying the restoration of natural vegetation have yet to be elucidated, as have its interactions with various abiotic factors, including soil salinity, erosion processes, and fluctuating groundwater levels. It remains largely unknown which plant species will dominate under these conditions and how species composition will evolve over the long term. Another key issue is the capacity of floodplain ecosystems to recover following a reduction in water levels and the disruption of the natural flood regime, as many species of floodplain flora depend on seasonal fluctuations in the water balance (Zhukov & Kunakh, 2025). The absence of a stable water regime can lead to the degradation of moisture-loving ecosystems, which more xerophytic communities may replace. The spread of invasive species, which can displace native flora and alter plant community structure, particularly on unstable soils, remains a significant concern. There are uncertainties regarding the rate at which new habitats develop and whether the lost wetlands will be replenished or replaced. Of equal concern is the impact of the altered hydrological regime on steppe ecosystems in surrounding areas. Declining groundwater levels may result in ecosystem degradation and aridification, altering both species composition and the functional organization of phytocoenoses (Burandt et al., 2023). The formation of new habitats in the aftermath of the Kakhovka reservoir disaster is fraught with significant uncertainties that necessitate long-term research to understand vegetation change patterns and develop effective environmental restoration measures.

The assessment of changes in the biota of floodplain ecosystems following the catastrophic destruction of a reservoir necessitates the use of practical ecological approaches (Gleick, Vyshnevskyi & Shevchuk, 2023), one of which is the concept of hemeroby (Ponomarenko et al., 2024). The concept of hemeroby, originally introduced by Jalas (Jalas, 1955) and further developed by Sukopp (1966), Sukopp (1971), Sukopp (1974), Kowarik (1988) and Kowarik (2019), has become a widely accepted indicator of human impact in vegetation science and landscape ecology. It reflects the degree to which plant communities are influenced by past and present anthropogenic disturbances that prevent the attainment of a potential natural state. While initially developed in the context of Central European flora, the concept has since been applied in diverse ecological settings. Recent research by Erdős et al. (2022) highlights the conceptual and methodological distinctions between hemeroby and naturalness, underscoring that hemeroby captures human-induced transformations at the level of plant communities and ecosystems, rather than merely characterizing individual sites. This broader interpretation supports the application of hemeroby in assessing functional and structural changes in vegetation under conditions of catastrophic disturbance. Applying the hemeroby concept enables the determination of the extent of changes in the flora and fauna of floodplain ecosystems. Hemeroby serves as an ecological indicator of the importance of anthropogenic transformation in ecosystems. It enables the classification of territories according to the level of human disturbance, ranging from natural habitats to those highly altered by human activity (Erdős et al., 2022). The destruction of the Kakhovka dam can be regarded as a sudden and significant anthropogenic impact that led to a rapid transition of floodplain ecosystems to higher levels of hemeroby (Spears et al., 2024). Before the disaster, most of the floodplain maintained natural vegetation and rich biodiversity (Skobel et al., 2023). The dam’s destruction left extensive areas drained or flooded, severely disrupting natural communities. The practical application of hemeroby for assessment can be achieved through the mapping of altered ecosystems, utilising the hemeroby scale to identify areas with varying levels of anthropogenic impact, and monitoring biodiversity by comparing biotic changes before and after the disaster (Komlyk, Ponomarenko & Zhukov, 2024). This approach will enable the identification of priority areas for environmental restoration, including the preservation of regions with the lowest levels of degradation and the restoration of natural biota habitats.

Our study aimed to investigate the syntaxonomic diversity of plant cover in the floodplain ecosystems of Khortytsia Island, shaped by the long-term impact of the Kakhovka Reservoir, and to assess the vegetation shifts resulting from its recent destruction. Additionally, we sought to evaluate the effects of altered hydrological conditions and related environmental gradients, as reflected in phytoindication scales such as soil moisture (Hd), moisture variability (fH), soil reaction (Rc), salinity (Sl), carbonate content (Ca), nitrogen availability (Nt), aeration (Ae), and climatic factors including temperature regime (Tm), ombroregime (Om), continentality (Kn), cryoregime (Cr), and light exposure (Lc), on the anthropogenic transformation of plant communities, which were assessed using the hemeroby index.

## Materials and Methods

### Study area

The Dnipro Hydroelectric Power Plant (HPP) near Zaporizhzhia was the first hydroelectric power station constructed on a river. Upon its completion in the 1930s, it was the largest hydroelectric power plant in Europe, with a capacity of 560 megawatts (MW). In August 1941, the retreating Red Army destroyed the dam; however, the plant was rebuilt after World War II. The second hydroelectric dam to be constructed was the Kakhovka Dam, which was completed in 1956. This dam is located downstream of the Dnipro HPP and approximately 60 kilometres upstream of Kherson. The Kakhovka Dam created the largest reservoir on the Dnipro River, significantly surpassing the reservoir created by the Dnipro HPP, with a volume of 18.2 cubic kilometres (km^3^) of water at a normal retaining level of 16.0 m (m) above mean sea level (MSL). In addition to generating hydropower, the primary purpose of the Kakhovka reservoir was to provide reliable irrigation and water supply to southern Ukraine. In the 1980s, the Zaporizhzhia Nuclear Power Plant (NPP), the most powerful in Europe, was constructed on the south bank of the Kakhovka reservoir near Enerhodar (Gleick, Vyshnevskyi & Shevchuk, 2023). The Kakhovka reservoir belonged to the Lower Dnipro sub-basin, which covers an area of 82,625 km^2^, accounting for approximately 28% of the total area of the Dnipro basin and 13.7% of the total area of Ukraine. The Dnipro River flows through this sub-basin for a total length of about 440 km, traversing the Dnipro, Zaporizhzhia, and Kherson regions of Ukraine (Iaroshevych et al., 2021). Kakhovka HPP was under the occupation of the Russian Federation. The Kakhovka dam and hydroelectric power plants were destroyed due to a massive explosion of the Kakhovka dam on 6 June 2023. The water level in the Dnipro River decreased by 8 m after the dam was destroyed, according to the Nikopol hydrological gauging station (Gleick, Vyshnevskyi & Shevchuk, 2023).

**Figure 1:**
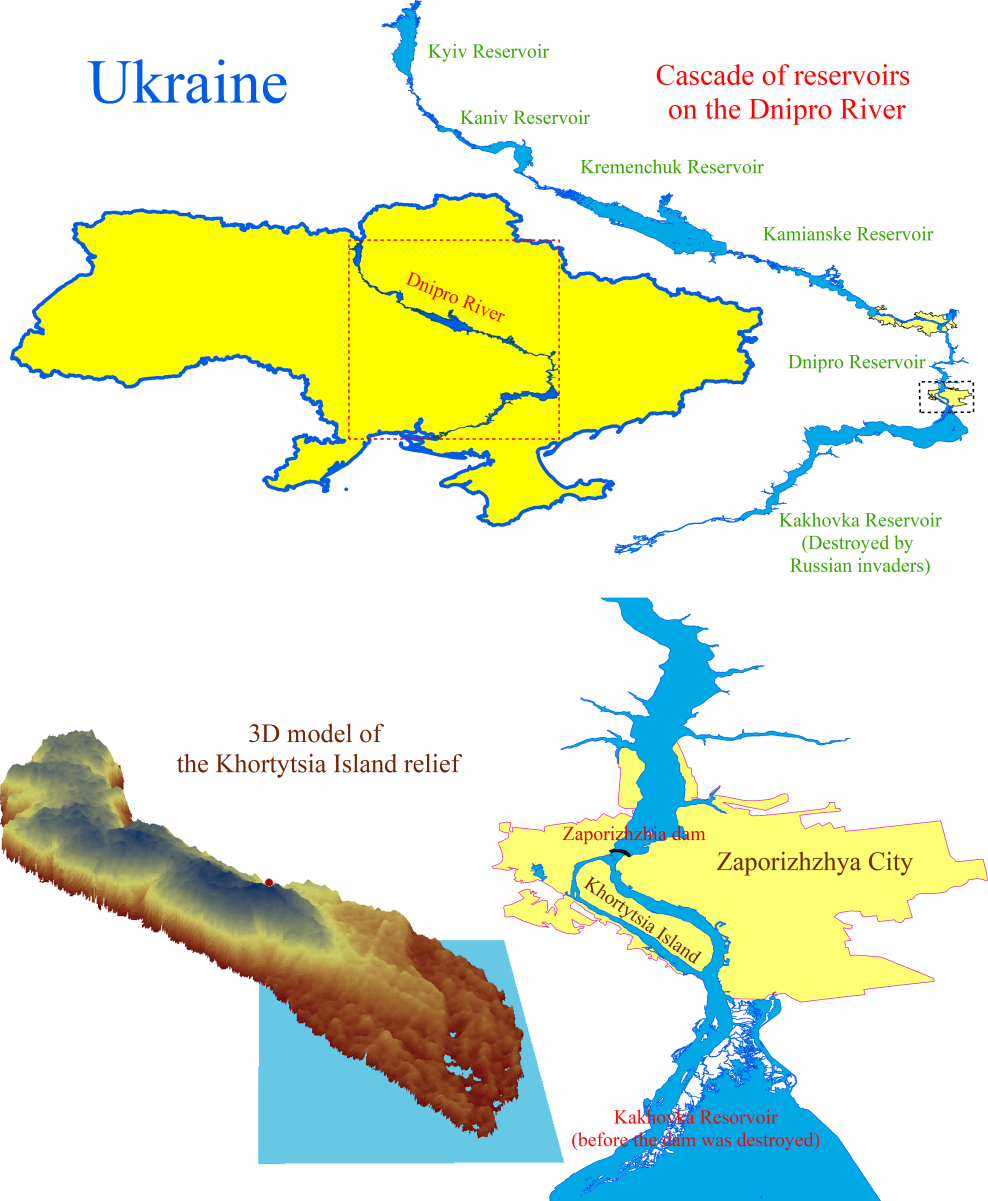
The cascade of reservoirs on the Dnipro River and the location and digital model of the Khortytsia Island. The blue quadrangle on the digital model of the island indicates the study area.

Khortytsia is the largest island on the Dnipro River, near Zaporizhzhia, downstream of the Dnipro hydroelectric power station (Fig. 1). The island extends from northwest to southeast, measuring 12 kilometres in length and 1.6 to 2.7 kilometres in width, encompassing an area of 2,359 hectares before the destruction of the Kakhovka dam. From a geological perspective, Khortytsia Island belongs to the Ukrainian crystalline shield, a Precambrian geological formation underlying much of central and southern Ukraine. The island’s geological foundation consists of a sloped granite plateau, which is covered by a layer of younger sedimentary rocks of varying thickness (Liashenko et al., 2022). The granite base rises 20 to 30 m above the river’s water surface in the island’s northern region. The elevation ranges from four to 15 m in the central area, while it lies below the water level in the southern (floodplain) section. The island’s territory can be categorised into three parts based on the predominant landforms. The rocky section is situated in the north, the upland area occupies the central part, and the lowland region is found at the southern edge, where floodplain ecosystems develop. Before the Kakhovka Dam’s destruction, the reservoir’s water level near Khortytsia Island was 15 m above sea level. The island’s northern part features a plateau that rises 58 to 62 m above sea level, culminating in granite cliffs. The central section of the island is the highest, with elevations reaching 75 to 77 m above sea level. The shoreline in this area is significantly indented due to an extensive system of gullies. In the southern part of the island, the elevation ranges from 17 to 35 m above sea level, and numerous floodplain lakes of varying sizes and depths are present. The flora of Khortytsia Island comprises 1,128 species of higher vascular plants, including 847 species of native flora and 281 cultivated species (Okhrimenko & Shelegeda, 2016).

### Descriptions of plant communities

The field study was conducted in the southern part of Khortytsia Island, covering a total area of approximately 5,300 m^2^. The research focused on two distinct landscape zones that were most affected by the destruction of the Kakhovka Reservoir: (1) floodplain lakes located within the interior of the island, and (2) the external shoreline along the Dnipro River, where the water level had dropped drastically. These zones were selected due to their pronounced hydrological alteration and ecological sensitivity. A total of 135 phytosociological relevés were recorded. Sampling plots were positioned to capture the variability of vegetation types across both former aquatic and transitionally terrestrial habitats. The selection of plots was stratified based on visible topographic and hydrological heterogeneity. Fieldwork was carried out between 10 and 21 August 2024, a period corresponding to the peak seasonal development of most invasive and ruderal plant species typical for this region. While certain early spring ephemeral species (*e.g.*, geophytes) may not be detected during this period, summer sampling is generally sufficient to assess the dominant community structure and hemeroby levels in floodplain systems. Instantaneous soil moisture values can vary over the course of the summer; however, in this study we used phytoindication-based indices, which reflect the long-term soil moisture regime as interpreted from the ecological preferences of the recorded species. The Braun-Blanquet approach to floristic classification was employed (Braun-Blanquet, 1964; Westhoff & Van Der Maarel, 1978). The standard size of plots was four × four m. The entire database of geobotanical relevés was processed and categorised into smaller groups based on their floristic differences using the two-way indicator species analysis (TWINSPAN) method (Hill, 1979) with the TWINSPAN for Windows 2.3 software (Hill & Šmilauer, 2005). The cut levels for “pseudospecies” were set at 0%, 2%, 5%, 10%, and 20%. Diagnostic species were identified within syntaxa using the fidelity index (Willner, Tichý & Chytrý, 2009). Pearson’s *phi* coefficient was used to determine the association between species and vegetation types (Chytrý et al., 2002). The *phi* coefficient was adjusted to account for differences in the number of sites among the plant community groups (Tichy & Chytry, 2006). The primary occurrence data from vegetation surveys conducted on Khortytsia Island and adjacent areas after the Kakhovka Reservoir disaster have been published in the Global Biodiversity Information Facility (GBIF) and are openly available at https://doi.org/10.15468/rewgwn (Zhukov et al., 2025).

### Phytoindication evaluation of ecological regimes

The calculation of environmental parameters was conducted using phytoindication scales. The scale developed by Didukh (2011) enables ordination analysis based on 12 factors: soil humidity (Hd), soil moisture variability (fH), soil aeration (Ae), soil nitrogen content (Nt), soil acidity (Rc), salt regime (Sl), carbonate content (Ca), temperature regime (Tm), ombroregime (Om), climate continentality (Kn), cryoregime (Cr), and light intensity (Lc).

The plant ecological groups are categorised into 23 gradations based on their relationship to the humidity regime (Didukh, 2011). The moisture regime scores can be converted into phytoindication estimates of the productive moisture stock within the one-meter soil layer, as outlined by Maslikova (2018a) and Molozhon et al. (2023):

\begin{eqnarray*}W=18.65\exp \nolimits (0.15H), \end{eqnarray*}

where *W* represents the content of productive moisture in the one-meter layer of soil (measured in millimetres), and *H* indicates a score of the moisture regime. A productive moisture content of less than 60 mm in a one-meter layer is classified as very low; a content ranging from 60 to 90 mm is classified as low; a content between 90 and 130 mm is deemed satisfactory; a content ranging from 130 to 160 mm is considered good; and a content exceeding 160 mm is classified as very good for agricultural plants (Vadunina & Korchagina, 1986).

The plant ecological groups were categorised into 12 gradations based on their relationship to the soil moisture variability. These scores can be converted into the coefficient of irregularity of soil moisture (*ω*), which ranges from 0 (indicating the lowest level of contrast in moisture conditions, such as consistently moist or consistently dry habitats) to 0.5 (indicating the highest level of contrast in moisture conditions, where nearly complete water immersion is followed by drought). The indicator scores of soil moisture variability can be converted to the moisture irregularity coefficient as follows (Maslikova, 2018b):

(1) \begin{eqnarray*}\omega =0.042fH-0.032,\end{eqnarray*}

where *ω* is the coefficient of irregularity of soil moisture, and *fH* is an indicator score of soil moisture variability.

The plant ecological groups were categorised into 15 gradations based on their relationship to the aeration regime. These scores can be converted into the percentage of air-filled porosity relative to the total porosity volume as follows (Maslikova, 2018b):

\begin{eqnarray*}P= \frac{100-A{e}^{4}}{100\ast (A{e}^{4}+1700)} , \end{eqnarray*}

where *P* represents the air-filled porosity percentage, which is the percentage of the total volume of pore space in the soil, and *Ae* denotes the score of the aeration regime.

The plant ecological groups are categorised into 15 gradations based on their tolerance to soil acidity. These scores can be converted to the pH of the soil aqueous solution as follows (Maslikova, 2018c):

(2) \begin{eqnarray*}\mathrm{pH}=2.26\mathrm{ln}(Rc)+1.88,\end{eqnarray*}

where pH is defined as the negative logarithm of the concentration of hydrogen ions in the soil solution, while *Rc* represents the acidity score.

The plant ecological groups are categorised into 19 gradations based on their relationship to the overall salt regime. These gradations can be converted into water-soluble salt content as follows (Maslikova, 2018c):

\begin{eqnarray*}S={2}^{0.6Sl+1}, \end{eqnarray*}

where *S* represents the salt content in the soil solution, measured in micrograms per litre (µg/l), and *Sl* denotes the score of the soil salinity regime.

The plant ecological groups were categorised into 13 gradations based on their response to the carbonate content of the soil. The scores can be converted to CaO + MgO content as follows (Maslikova, Zhukov & Kovalenko, 2019):

\begin{eqnarray*}\mathrm{CaO}+\mathrm{MgO}=14 \frac{C{a}^{4.5}}{C{a}^{4.5}+45000} , \end{eqnarray*}

where CaO+MgO is the carbonate content, expressed in terms of calcium and magnesium oxides (%). *Ca* serves as a score for the carbonate content.

The plant ecological groups are categorized into 11 gradations based on their relationship to soil nitrogen content. The scores can be converted to soil nitrogen content as follows (Maslikova, Zhukov & Kovalenko, 2019):

\begin{eqnarray*}N=5 \frac{N{t}^{3.7}}{N{t}^{3.7}+345} , \end{eqnarray*}

where *N* represents the soil’s nitrogen content, measured in grams per kilogram (g/kg), and *Nt* is a score that indicates the soil’s trophic regime.

The plant ecological groups are categorised into 17 gradations based on their relationship to the thermal regime. These scores can be translated into a radiation balance as follows (Molozhon et al., 2023):

(3) \begin{eqnarray*}RB=0.21Tm,\end{eqnarray*}

where *RB* is the radiation balance measured in *gJ* m–^2^ year^−1^, while *Tm* represents the thermal regime score, one must note that if the radiation balance is expressed in Kcal cm^−2^ year^−1^, one can multiply the score by 5.

The plant ecological groups are classified into 23 categories based on their relationship to atmospheric humidity regimes. Ombroclimate scores can be interpreted as the difference in the amount of precipitation before evaporation from open water surfaces, measured annually in terms of average days (Molozhon et al., 2023):

(4) \begin{eqnarray*}Hum=0.54Om-7,\end{eqnarray*}

where *Hum* represents the difference between the average daily precipitation and the evaporation from the open water surface over the same period, measured in millimetres (mm). *Om* denotes the climate humidity score.

The plant ecological groups are categorized into 17 gradations based on their relationship to continentality. The continentality scores can be converted to the Ivanov continentality scale (Ivanov, 1959) as follows (Molozhon et al., 2023):

(5) \begin{eqnarray*}SKn=10~Kn+41,\end{eqnarray*}

where *SKn* represents the Ivanov continentality scale, *Kn* denotes the score of the continentality regime.

The plant ecological groups are divided into 15 gradations based on their relationship to the cryoclimate. The cryoclimate scores can be converted to reflect the average temperature of the coldest month of the year as follows (Molozhon et al., 2023):

(6) \begin{eqnarray*}Temp=3.83Cr-38.17,\end{eqnarray*}

where *Temp* represents the average temperature of the coldest month of the year, measured in degrees Celsius (^∘^C), while Cr is a score that indicates the cryoclimate.

The relationship between the measured light and the photoinduction assessment of light levels is as follows:

(7) \begin{eqnarray*}\text{log_Lighiting}=0.22\ast L\text{-value},\end{eqnarray*}

where log_Lighiting is the decimal logarithm of the relative light level, *L-*value is the phytoindication assessment of the light level according to the Ellenberg scale.

### Geomorphological calculations

The configuration of the water bodies was reconstructed using detailed satellite imagery from Bing Maps (https://www.bing.com/maps) and further refined through field surveys. The depth map of the Dnipro riverbed was derived from Navionics SonarChart™ data (http://www.navionics.com). A digital elevation model (DEM) of the surface area was created based on data obtained from the Advanced Land Observation Satellite (ALOS) (https://www.eorc.jaxa.jp/ALOS/en/index_e.htm). The spatial resolution of this model is 12.5 m. The morphometric characteristics of the reservoirs were calculated using the *lakemorpho* (Hollister & Stachelek, 2017).

## Results

### Catastrophic dynamics of morphometric parameters of floodplain water bodies

**Figure 2:**
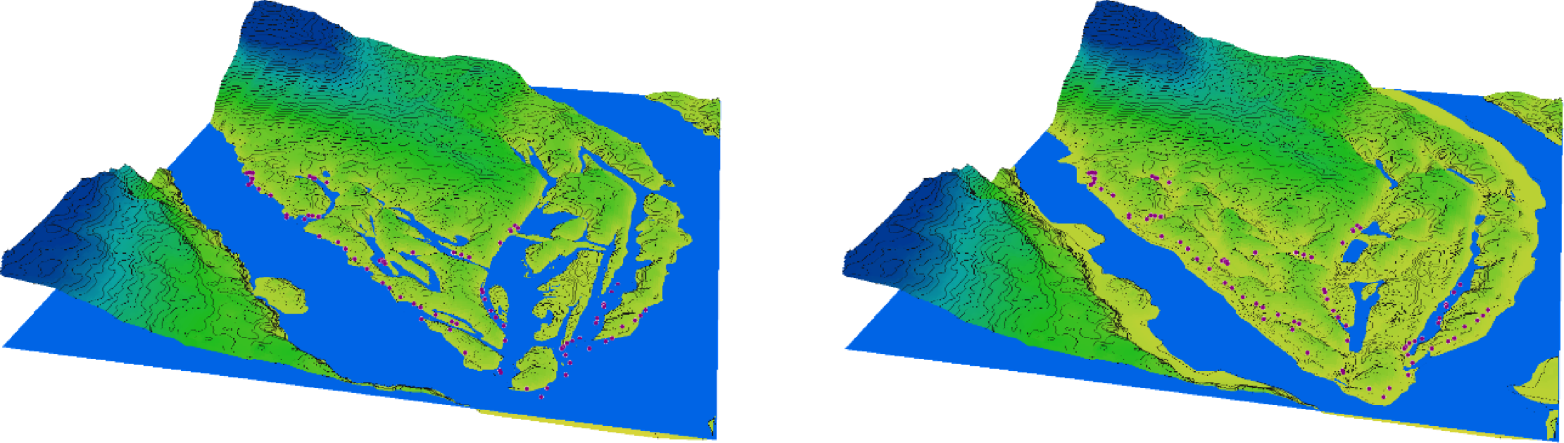
3D diagram of the terrain in the southern part of Khortytsia Island and the water level in the Dnipro River in late summer before the destruction of the Kakhovka Dam (2022) and after its destruction (2024).

In the period preceding the disaster, the natural decline in water levels that occurred after the regular spring flood and continued into the summer led to a significant reduction in the area of floodplain water bodies on Khortytsia Island (Fig. 2). This decline also affected the shoreline length, surface area, and volume of the floodplain water bodies, though the extent of reduction varied across parameters (Table 1). Following the destruction of the Kakhovka Dam, the spring values of shoreline length, surface area, and water volume decreased by 76.8%, 85.6%, and 79.9%, respectively, compared to the corresponding spring measurements before the disaster. During the summer period, these reductions were 79.2%, 88.4%, and 79.3%, respectively. The seasonal (spring-to-summer) decrease in shoreline length after the disaster was 23.1%, which exceeded the pre-disaster seasonal decrease of 14.4%. Similarly, the summer reduction in surface area reached 41.2% after the disaster, compared to 26.7% before. In contrast, the seasonal change in water volume remained relatively stable, at 7.0% post-disaster *versus* 9.3% prior to the event.

**Table 1:** Changes in the morphometric parameters of floodplain water bodies in the southern part of Khortytsia Island.

Parameters	Before the disaster			After the disaster			Δ Spring- Spring, %.	ΔSummer- Summer, %.
	Spring	Summer	Δ, %	Spring	Summer	Δ, %		
Shoreline length, m	38,658	33,073	14.4	8,953	6,882	23.1	76.8	79.2
Surface area, m^2^	1,929,062	1,413,644	26.7	278,267	163,523	41.2	85.6	88.4
Volume, m^3^	61,222,951	55,537,364	9.3	12,330,116	11,469,570	7.0	79.9	79.3

### Structure of the vegetation cover

The surveys identified 146 plant species. On average, each survey recorded 12.1 ± 4.6 species, ranging from 5 to 28. The projected cover of grass and shrub vegetation was 50.8 ± 23.7%, varying from 3% to 97%. The analysis identified 12 distinct plant associations.

**Table:** 

*Bidentetea* tx. et al. ex Von Rochow 1951
*Bidentetalia* Br.-Bl. et Tx. ex Klika et Hadač 1944
*Bidention tripartitae* Nordhagen ex Klika et Hadač 1944
*Leersio-Bidentetum* (Koch 1926) Poli et Tx. 1960
*Chenopodion rubri* (Tx. in Poli et J. Tx. 1960) Hilbig et Jage 1972
*Chenopodietum rubri* Timár 1950
*Isoëto-Nanojuncetea* Br.-Bl. et Tx. in Br.-Bl. et al. 1952
*Nanocyperetalia* Klika 1935
*Eleocharition soloniensis* Philippi 1968
*Cyperetum micheliani* Horvatić 1931
*Phragmito-Magnocaricetea* Klika in Klika et Novak 1941
*Phragmitetalia* Koch 1926
*Phragmition communis* Koch 1926
*Phragmitetum australis* Savič 1926
*Typhetum angustifoliae* Pignatti 1953
*Artemisietea vulgaris* Lohmeyer et al. in Tx. ex von. Rochow 1951
*Onopordetalia acanthii* Br.-Bl. et Tx. ex Klika et Hadač 1944
*Onopordion acanthii* Br.-Bl. et al. 1936
*Xanthietum strumarii* Paucaˇ 1941
*Salicetea purpureae* Moor 1958
*Salicetalia purpureae* Moor 1958
*Salicion albae* Soó 1951
*Salicetum albae* Issler 1926
*Salici-Populetum* (Tx. 1931) Meijer-Drees 1936
*Stellarietea mediae* Tx. et al. in Tx. 1950
*Atriplici-Chenopodietalia albi* (Tx. 1937) Nordhagen 1940
*Amarantho blitoidis-Echinochloion cruris-galli* Solomakha 1988
*Amarantho retroflexi-Echinochloetum cruris-galli* Bagrikova 2005
*Eragrostietalia* J. Tx. ex Poli 1966
*Eragrostion* Tx. in Oberd. 1954
*Portulacetum oleracei* Felföldy 1942
*Sisymbrietalia sophiae* J. Tx. ex Görs 1966
*Sisymbrion officinalis* Tx. et al. ex von Rochow 1951
*Erigeronto-Lactucetum serriolae* Lohmeyer in Oberd. 1957
*Salsolion ruthenicae* Philippi ex Oberd. 1983
*Bromo tectorum-Corispermetum leptopteri* Sissingh et Westhoff ex Sissingh, 1950 corr. Dengler 2000

**Figure 3:**
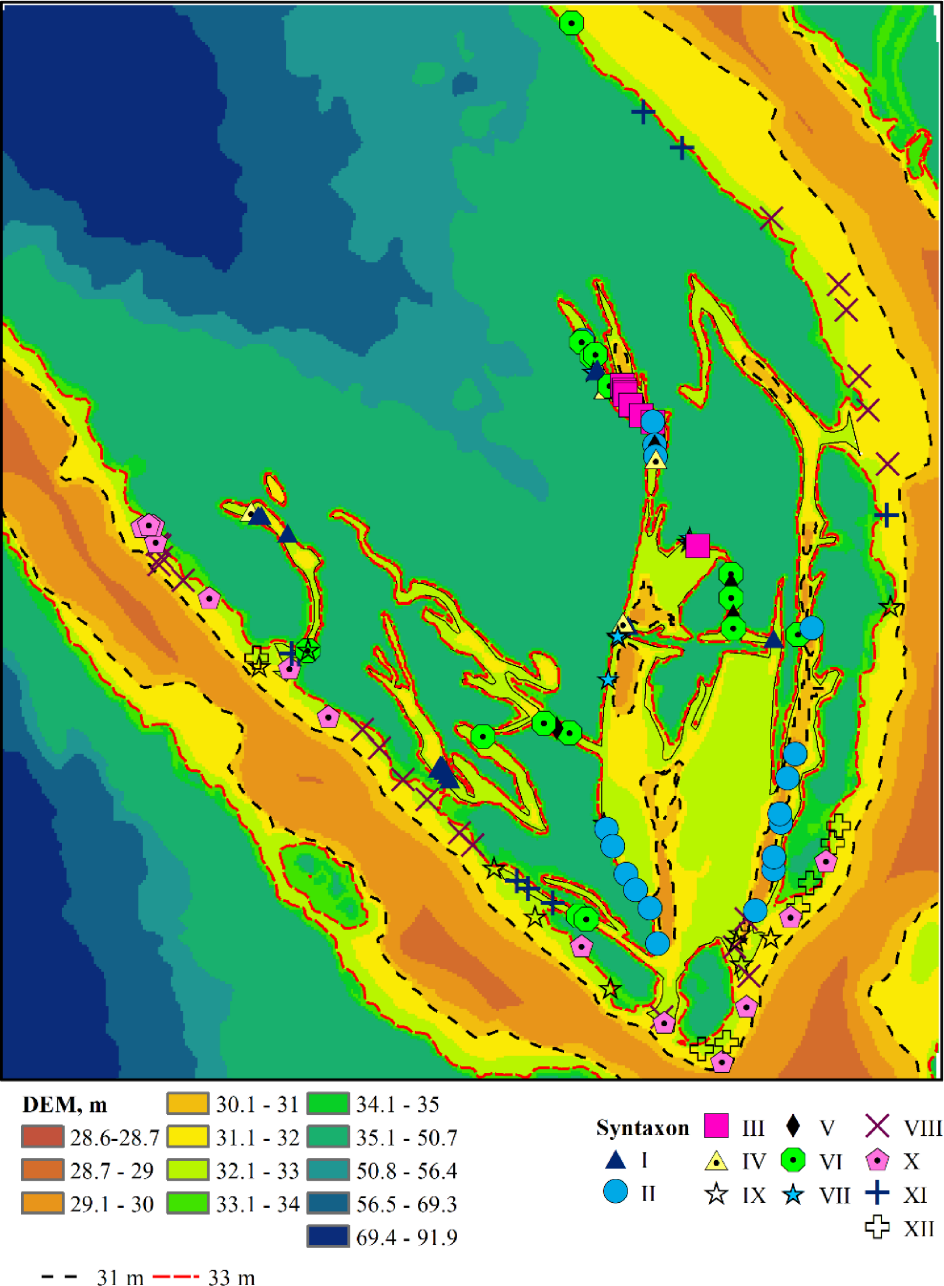
Plant associations and their spatial location. I–*Leersio-Bidentetum* (Koch 1926) Poli et Tx. 1960; II–*Chenopodietum rubri* Timár 1950; III–*Cyperetum micheliani* Horvatić 1931; IV–*Phragmitetum australis* Savič 1926; V–*Salicetum albae* Issler 1926; VI–*Salici-Populetum* (Tx. 1931) Meijer-Drees 1936; VII–*Typhetum angustifoliae* Pignatti 1953; VIII–*Portulacetum oleracei* Felföldy 1942; IX–*Xanthietum strumarii* Paucaˇ 1941; X–*Amarantho retroflexi-Echinochloetum cruris-galli* Bagrikova 2005; XI–*Erigeronto-Lactucetum serriolae* Lohmeyer in Oberd. 1957; XII–*Bromo tectorum-Corispermetum leptopteri* Sissingh et Westhoff ex Sissingh, 1950 corr. Dengler 2000. The black and red dashed lines indicate the water levels in the Dnipro River before and after the destruction of the Kakhovka Dam in 2023.

The associations are numbered according to the order in which they were identified by the TWINSPAN procedure (Table S1), reflecting the most significant environmental gradient along which the plant communities are organised. Humidity serves as this gradient, as indicated by the estimates of the humidity regime (Table S2). The associations comprising *Leersio-Bidentetum*, *Chenopodietum rubric*, *Cyperetum micheliani*, and *Typhetum angustifoliae* are characterized by the highest moisture levels. These associations represent pioneering communities of tall annuals found in waterlogged areas and anthropogenically disturbed, often nitrified and silted ecotopes. They include communities of hygrophilous annuals with ephemeral vegetation in regions experiencing sharp fluctuations in moisture levels and communities of tall aquatic macrophytes, whose lower parts are predominantly submerged in water during the growing season. These communities are distinguished by high projective cover and species richness. *Leersio-Bidentetum* associations are located in floodplain reservoirs that are relatively distant from the island’s bank (Fig. 3). *Chenopodietum rubric* associations are typical of the main channels within the island. The *Cyperetum micheliani* association is found in the most remote water bodies of the island*. Typhetum angustifoliae* associations occupy transitional positions between the most distant water bodies and those directly connected to the main channel of the Dnipro River.

**Figure 4:**
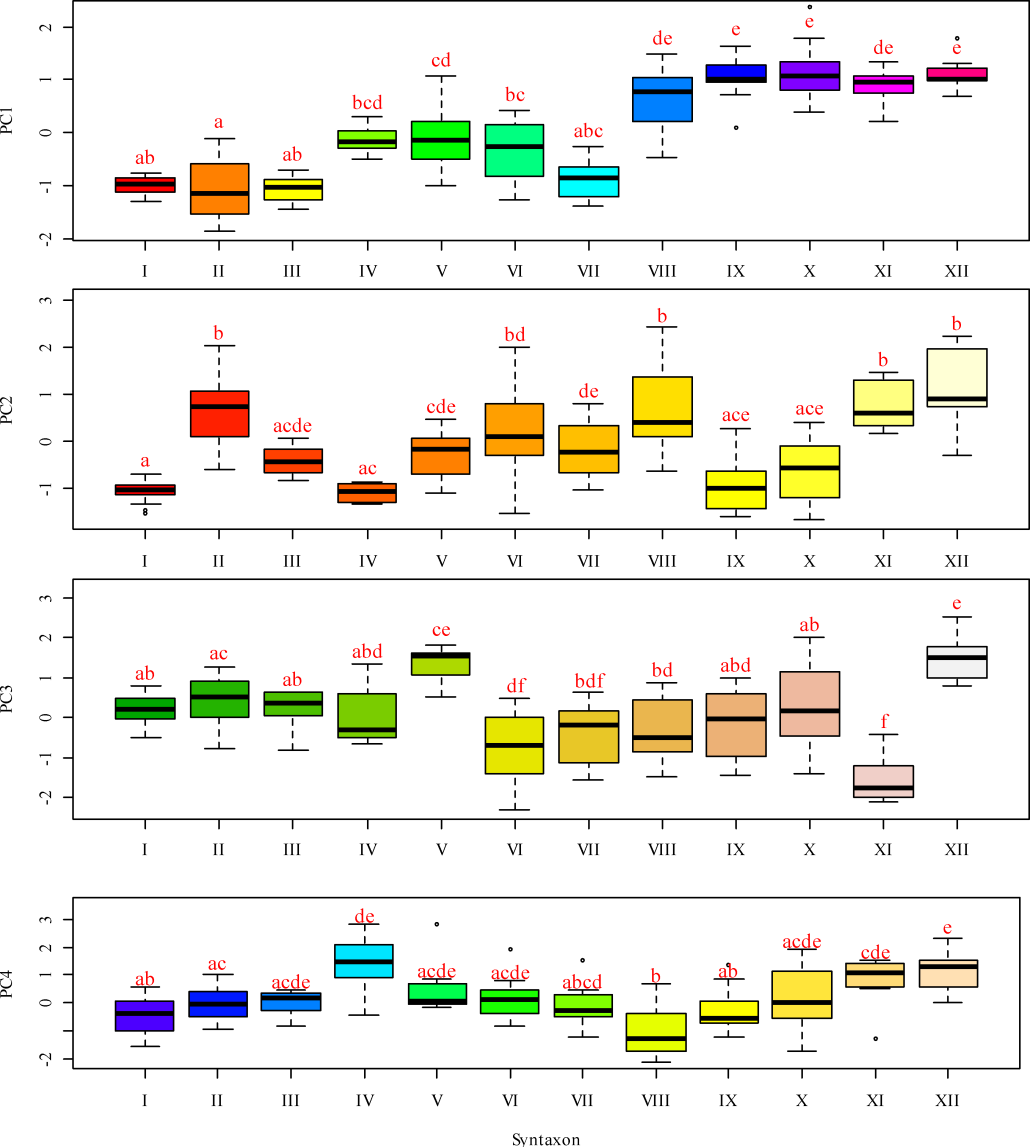
Box-and-whiskers plots illustrating the variation in PC 1 through FC 4 scores across different functional traits. The ordinate represents the scores for Fide 1 to Fide 5, while the abscissa delineates the following categories: I–*Leersio-Bidentetum* (Koch 1926) Poli et Tx. 1960; II–*Chenopodietum rubri* Timár 1950; III–*Cyperetum micheliani* Horvatić 1931; IV–*Phragmitetum australis* Savič 1926; IX–*Xanthietum strumarii* Paucaˇ 1941; V–*Salicetum albae* Issler 1926; VI–*Salici-Populetum* (Tx. 1931) Meijer-Drees 1936; VII–*Typhetum angustifoliae* Pignatti 1953; VIII–*Portulacetum oleracei* Felföldy 1942; X–*Amarantho retroflexi-Echinochloetum cruris-galli* Bagrikova 2005; XI–*Erigeronto-Lactucetum serriolae* Lohmeyer in Oberd. 1957; XII –*Bromo tectorum-Corispermetum leptopteri* Sissingh et Westhoff ex (Sissingh, 1950) corr. Dengler 2000. The box plot displays the interquartile range, which encompasses the first quartile (Q1, 25%) and the third quartile (Q3, 75%), with the median (Q2, 50%) represented by the line within the box. The whiskers indicate the minimum and maximum values that are not classified as outliers. According to the results of the Dunn test, identical letters denote levels of factors that are statistically indistinguishable at a significance level of *P* < 0.05, while differing letters indicate levels of factors that are statistically significantly different.

A lower moisture level was observed in the *Salicetum albae* and *Salici-Populetum* associations (Fig. 4). These communities consist of floodplain and riverbank willow and poplar forests and shrubs, which experience varying flood durations on slightly silted sandy and silty soils, with a proximity to fresh groundwater. The *Phragmitetum australis* is a transitional vegetation type in terms of moisture levels between these groups of associations. This community thrives in eutrophic freshwater and brackish low-flow or closed water bodies, characterised by a neutral to slightly alkaline pH. During the growing season, it undergoes substantial fluctuations in water levels, typically ranging from 25 to 50 cm, and occasionally reaching up to 70 cm. These fluctuations are associated with bottom sediments composed of sandy-silt, silt, silty-peat, and peat. It also exhibits dense plant cover and high species richness. The *Salicetum albae* association is typically found in dried-up channel beds that connect floodplain reservoirs. The *Salici-Populetum* associations are close to one another and share ecologically similar environmental conditions. A notable feature of the spatial distribution of *Salici-Populetum* associations, compared to *Salicetum albae* associations, is their tendency to be found more frequently in wider or dried lake-type channels that were once characterised by significant water flow. When these two associations occur adjacent, *Salici-Populetum* associations are likelier to thrive on local micro-uplands or in the upper portions of the thicket belt along the water’s edge before it dries up. Conversely, *Salicetum albae* associations are commonly found in local micro-depressions or closer to the bottom of a dried-up pond. Ecologically, *Salicetum albae* associations prefer habitats with higher nutrient content and lower pH levels than those favoured by *Salici-Populetum* associations. *Phragmitetum australis* associations are frequently found in desiccated watercourses and streams, particularly in local depressions in the landscape where moisture accumulates.

Other associations represent plant communities that tolerate drier conditions while experiencing greater variability in moisture availability. Notably, attention should be drawn to the *Erigeronto-Lactucetum serriolae* association, which is characterised by a predominance of more neutral soil pH levels, a higher concentration of soluble salts in the soil, and the highest degree of soil aeration. These plant communities thrive in compacted, nitrified, and loose substrates and are commonly found along clearings in deciduous forests or on previously ploughed river floodplains. The *Xanthietum strumarii* association is distinguished by its highest cryoclimate score. In contrast, the *Bromus tectorum-Corispermum hyssopifolium-associatie* association prefers conditions characterised by very low cryoclimate scores. This association is frequently found in anthropogenically disturbed areas, such as roadsides, railway embankments, and dry riverbeds. The *Portulacetum oleracei* association primarily inhabits steep, sandy banks devoid of water located along the island’s outer edge. The *Xanthietum strumarii* association occupies similar positions but is typically found in areas with more level terrain, occasionally at the bottoms of dried-up water bodies. The *Amarantho retroflexi-Echinochloetum cruris-galli* association is commonly located on the sandy banks in the southeastern or southern parts of the island. The *Bromus tectorum-Corispermum hyssopifolium-associatie* association is most frequently observed in the island’s southeast region.

### Principal component analysis of variability in phytoindication-based assessments of environmental factors

**Figure 5:**
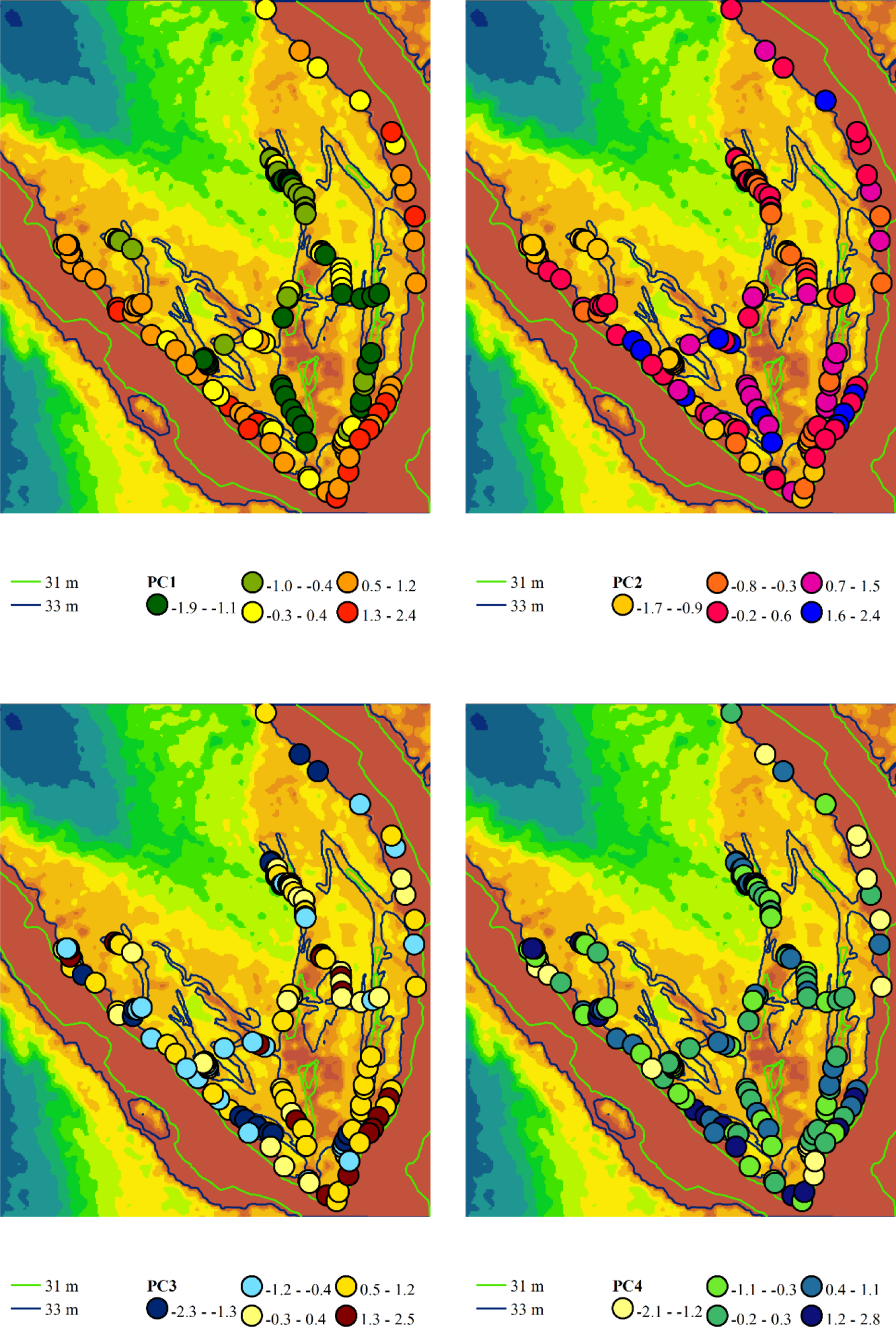
Spatial variation of the principal component scores extracted from the variability analysis of phytoindication scales. Principal component 1 (PC1) represents the gradient of moisture and hemeroby, where negative scores indicate higher moisture and lower hemeroby, while positive scores indicate lower moisture and higher hemeroby. Principal component 2 (PC2) reflects the gradient of soil nitrogen content and hemeroby, with negative slopes corresponding to higher nitrogen content and greater hemeroby, and positive slopes indicating lower nitrogen content and reduced hemeroby. Principal component 3 (PC3) illustrates the gradient of light and hemeroby, where negative scores signify lower light levels and reduced hemeroby, while positive scores denote higher light levels and lower hemeroby. Principal component 4 (PC4) represents the gradient of soil particle size distribution, with negative scores indicating sandy, carbonate-free soils, and positive scores indicating soils with higher clay and carbonate content.

Principal component analysis identified four components with eigenvalues exceeding one, in accordance with Kaiser’s criterion (Table 2). Principal component 1 accounted for 32.5% of the variability in the feature space. This component exhibited the highest loadings on the moisture regime indicator scales, allowing it to be meaningfully interpreted as a moisture gradient. More humid conditions are typically associated with more natural plant communities, while more arid conditions are linked to hemeroby communities. Other ecological regimes also change naturally along this moisture gradient. As aridity increases, the variability of moisture, carbonate content of the soil, aeration regime, thermal regime, continental regime, cryoregime, and light regime also increase. Concurrently, with rising moisture content, nutrient levels and ombroclimate indicators generally increase. The lowest scores of principal component 1 were characteristic of near-water plant communities, whereas the highest scores were typical of sandy bank plant communities (Fig. 5).

**Table 2:** Principal component analysis of variability of environment, naturalness and hemeroby indicators (only factor weights that are statistically significant at *P* < 0.05 are displayed).

Variable		Principal component (eigenvalue *λ* and the percentage of the explained variance)			
		PC1, *λ* = 6.5, 32.5%	PC2, *λ* = 3.0, 15.0%	PC3, *λ* = 2.8, 13.7%	PC4, *λ* = 1.7, 8.7%
Didukh indicator scales	Hd	−0.92	–	–	–
	fH	0.78	0.33	–	–
	Rc	–	0.18	−0.50	0.66
	Sl	–	−0.28	−0.16	0.64
	Ca	0.32	−0.23	−0.26	0.62
	Nt	−0.45	−0.70	–	–
	Ae	0.78	0.25	−0.18	–
	Tm	0.43	−0.31	−0.36	−0.20
	Om	−0.72	−0.30	–	0.29
	Kn	0.56	0.28	–	0.32
	Cr	0.28	−0.47	−0.36	−0.43
	Lc	0.41	0.49	0.21	–
Ellenberg indicator scales	Light Regime	−0.21	0.17	0.85	–
	Temperatures	0.80	−0.31	−0.21	−0.22
	Continentality of Climate	0.66	−0.39	0.29	–
	Humidity	−0.90	–	–	–
	Acidity	0.34	0.40	−0.66	–
	Nutrients Availability	−0.12	−0.64	−0.55	–
Naturalness and hemeroby	Naturalness	−0.59	0.55	−0.47	–
	Hemeroby	0.66	−0.52	0.45	–

Principal component 2 accounted for 15.0% of the variability in the traits analysed. This component exhibited the highest loadings on indicator scores related to nutrient content, allowing it to be interpreted as a trophic gradient. Additionally, principal component 2 demonstrated significant loadings on indicators of naturalness and hemeroby within plant communities. An increase in trophicity corresponds with a rise in hemeroby, while a decrease in trophicity is associated with an increase in the naturalness of plant communities. The increase in trophicity is correlated with higher levels of soluble salts and carbonates in the soil, which serve as indicators of the thermoregime, ombroregime, and cryoregime. Conversely, a decrease in trophicity is linked to more significant variability in moisture, pH, and improvements in aeration, lighting, and continentality. The highest scores of principal component 2 were observed in the associations *Chenopodietum rubri* and *Typhetum angustifoliae*, while the lowest scores were found in *Erigeronto-Lactucetum serriolae* and *Amarantho retroflexi-Echinochloetum cruris-galli*.

Principal component 3 accounted for 13.7% of the variability in the feature space. This component exhibited the highest loading on the indicator score for the lighting regime and was significantly correlated with naturalness and hemeroby. The increase in hemeroby was associated with a rise in the light regime. At the same time, the growth of naturalness was characterised by elevated pH levels, carbonate and dissolved salt content, soil aeration, and indicators of thermal and cryoregime. The highest scores of principal component 3 were observed in the associations *Salicetum albae* and *Bromus tectorum-Corispermum hyssopifolium*, whereas the lowest scores were found in *Salici-Populetum* and *Erigeronto-Lactucetum serriolae*.

Principal component 4 explained 8.7% of the variability in the feature space. This component was not sensitive to the naturalness and hemeroby of the communities. It exhibited the highest loadings for pH, carbonate, and dissolved salt content in the soil. Therefore, this component can be interpreted as an indicator of the soil’s granulometric composition. The highest scores of this component were observed in the *Phragmitetum australis* and *Bromo tectorum-Corispermetum leptopteri* associations, while the lowest scores were found in the *Portulacetum oleracea* association.

## Discussion

The development of floodplain ecosystems is driven by alternating periods of flooding and drying, resulting in high moisture variability (Blom & Voesenek, 1996). The natural flow regime and its seasonal components are the primary driving forces behind the functions and processes of both abiotic and biotic elements, including morphology, water quality, floodplains, groundwater, riparian vegetation, fish, macroinvertebrates, and amphibians. Consequently, they play a crucial role in maintaining the integrity of floodplain river ecosystems (Hayes et al., 2018). The duration of flooding in large temperate rivers, such as the Dnipro, typically lasted between 30 and 40 days before regulation. Water levels could rise by several meters, presenting a significant extreme factor for both flora and fauna (Kozlowski, 2002). The ability of plants and animals to endure flooding is considered a crucial adaptation (Schindler et al., 2022). Flooding should be recognised as a critical hydrological process for clearing floodplains of sediments and excess organic matter, thereby maintaining a stable ecological balance in river systems (Jakubínský et al., 2021). The runoff of the Dnipro River is entirely managed by a series of reservoirs. The intra-annual runoff distribution in the lower reaches of the Dnipro has changed significantly. The reduction in annual runoff from 1,610 m^3^/s, prior to the damming of the Dnipro River by the reservoir cascade, to 1,310 m^3^/s can be attributed to increased water consumption, primarily for irrigation, evaporation from the surfaces of the reservoirs, and the volume of water needed for the initial filling of the reservoirs. The actual runoff of the Dnipro is approximately 20% lower than it would be under natural conditions (Iaroshevych et al., 2021). The construction of dams on rivers and the establishment of associated reservoir systems have significantly altered the hydrological regimes of these waterways (Nascimento do Vasco, De Oliveira Aguiar Netto & Gonzaga da Silva, 2019). The flooding phenomenon has nearly vanished following constructing a series of reservoirs on the Dnipro River. While there is a seasonal rise in water levels during the spring, the water level declines very slowly for the remainder of the year, resulting in a seasonal drop comparable to the daily fluctuations in the reservoir’s water levels (Zhukov et al., 2022). It can be argued that the creation of artificial reservoirs has prolonged the duration of floods, leading to changes in ecosystem conditions and structure. The sudden failure of a dam and the rapid decrease in water levels can trigger catastrophic events that initiate succession processes in plant community dynamics in the areas exposed as the water recedes. The extensive region formerly occupied by the Kakhovka Reservoir was significantly impacted. The floodplain ecosystems of Khortytsia Island serve as a valuable and relevant model site for studying the consequences of the reservoir’s destruction. This area is designated as a nature reserve and is characterised by a high biological and landscape diversity level.

The water volume in floodplain water bodies has decreased by an average of five times due to the destruction of the reservoirs, which has become a significant environmental factor. The impact of the lowered water level varies between the bank of Khortytsia Island, which is adjacent to the Dnipro River channel, and the floodplain water bodies in the island’s interior. A new land area along the island’s bank is now covered with sand. These biotopes are extreme, characterised by low nutrient levels, minimal organic matter, reduced dissolved salts, and considerable sand movement (Moreno-Casasola, 1986). The sand exhibits poor capillary properties (Hird & Bolton, 2017), leading to rapid drying and overheating (Dubey et al., 2021). Only highly specialised plant species can thrive in a harsh environment (Cao et al., 2024). Inland floodplain reservoirs have either wholly vanished or experienced significant reductions in area. It is also noteworthy that, despite the substantial decrease in water volume in the floodplain reservoirs, the variability in the length of the shoreline and the surface area of the reservoirs during summer, compared to spring, has increased relative to pre-disaster levels. This suggests that an increase in the variability of the moisture regime within floodplain ecosystems has accompanied the reduction in water volume in the water bodies.

The explosion of the Kakhovka Dam and the subsequent destruction of the Kakhovka Reservoir constituted a significant anthropogenic factor that profoundly impacted the living conditions of the biota within floodplain ecosystems. It is evident that the anthropogenic impact elicited varying responses across different types of ecosystems and at various levels of exposure to natural environmental factors. The proportion of variance explained by the principal components may seem moderate; however, this distribution reflects the ecological reality of the studied system. Notably, there is a stark contrast between the relatively small share of variation attributed to natural background factors and the predominant share explained by anthropogenically driven gradients. Principal components 1 to 3 collectively account for the majority of the total variance and are clearly associated with indices of hemeroby and naturalness, which serve as indicators of anthropogenic transformation. These components capture significant ecological shifts resulting from the altered hydrological regime, nutrient dynamics, and increased aridity following the destruction of the Kakhovka Reservoir. In contrast, principal component 4, which explains a smaller portion of the variance and is not linked to the hemeroby–naturalness gradient, represents more stable environmental factors such as soil pH, carbonate content, and granulometric properties. This distribution of explained variance underscores the predominance of recent anthropogenic pressures over intrinsic environmental variability in shaping vegetation patterns during the early stages of post-disturbance succession. The hemeroby index was proposed as a means to characterize the response to anthropogenic influences following dam destruction. The hemeroby index was proposed as a means to characterize the response of plant communities to anthropogenic influences induced by the destruction of the dam. This compound impact was triggered by the abrupt drop in water levels, with subsequent mobilization of toxic substances previously buried in the bottom sediments, as well as the spread of invasive species from adjacent agricultural and urban ecosystems into the newly exposed terrestrial habitats. These processes are ongoing, interact in complex ways, and collectively represent a multifactorial anthropogenic driver of vegetation change. Our findings indicate that three of the four principal components representing the primary natural trends in the variability of plant community structure were associated with the indices of hemeroby and naturalness. It can be inferred that the destruction of the reservoir triggered succession processes within the dynamics of plant communities. If we assume that the hemeroby and naturalness indices provide a quantitative representation of the catastrophic anthropogenic factor caused by the destruction of the reservoir, then the structure of the eigenvalues indicates the following pattern: the eigenvalues of the corresponding principal components decrease in order of the sensitivity of the respective community property complexes to the impact of this anthropogenic factor, while simultaneously increasing with respect to the component that reflects the stationary natural dynamics of plant communities. This assumption is supported by the observation that principal component 4, which accounts for a significantly smaller proportion of community variability, was not sensitive to hemeroby and the naturalness of plant communities. This indicates that the pattern represented by this component has a natural origin. Principal component 4 exhibited the highest loadings on the phytoindication estimation of pH, carbonate content, and nutrient levels, which may explain the changes in soil particle size distribution. Sandy alluvial soils typically lack carbonates and are low in nutrients. Thus, the increase in carbonate content, pH, and nutrient levels is likely a result of an increase in the clay and loam fractions within the soil particle size distribution. It is important to note that this interpretation also underscores the ’relict’ nature of the trend, as particle size distribution is a relatively conservative soil property (Filgueira et al., 2006).

Principal component 1 was found to be sensitive to hemeroby and naturalness. The significant loadings of this component on the environmental factors associated with moisture regimes suggest that it reflects changes in the community resulting from the destruction of the reservoir and the catastrophic alteration of the water regime in floodplain ecosystems. This principal component emphasizes that the increase in arid conditions is a significant driver of anthropogenic transformation within these communities, as reflected by the hemeroby values. This finding aligns with research indicating that a plant community’s resilience to disturbance events is contingent upon the characteristics of its water regime (Cole, 1995). The plant communities in the floodplain, where soil moisture levels are consistently high, exhibit greater naturalness. Spatially, principal component 1 indicates a significant anthropogenic transformation of plant communities along the island’s bank, particularly in the area adjacent to the Dnipro River channel. The river’s riparian zone, which has significantly expanded following the disaster, is inhabited by the plant communities that can withstand high levels of anthropogenic pressure, particularly specific associations *Erigeronto-Lactucetum serriolae*, *Amarantho retroflexi-Echinochloetum cruris-galli*, and *Bromo tectorum-Corispermetum leptopteri*. The association *Bromo tectorum-Corispermetum leptopteri* was previously described under the name *Bromus tectorum-Corispermum hyssopifolium-associatie* (Sissingh, 1950), but in 2002 it was made to correct the name in accordance with the rules of the International Code of Phytosociological Nomenclature (ICPN) (Dengler, 2002). This correction was re-presented in the article Dengler et al. (2003). The *Bromo tectorum-Corispermetum leptopteri* association is part of the thermophilic ruderal vegetation of Central Europe, which rapidly colonizes open, unstable areas but can be gradually supplanted by other formations during the process of natural succession. The primary species that define its composition are *Bromus tectorum* and *Corispermum hyssopifolium*, both of which are characteristic of dry, sandy, or rocky substrates. This association is unstable, pioneering, and xerophytic, primarily consisting of annual and ephemeral plants that undergo rapid changes in response to environmental conditions. The species within this community play a crucial role in the initial stages of succession following disturbances to natural vegetation. However, due to their inherent dynamism, such communities are typically transient and highly susceptible to modification by external environmental factors (Eliáš, 1982). The *Bromo tectorum-Corispermetum leptopteri* is a ruderal association that occurs on dry, sandy substrates and is composed of short-lived, rapidly changing plant communities (Dengler et al., 2003). This association represents a floristically diverse community that is characteristic of the early stages of succession dynamics. The herbaceous layer is predominantly composed of annual species. It is important to note that the presence of certain neophytes in this association, such as *Erigeron canadensis*, can be significant. Moss cover is minimal or entirely absent. This community thrives in loose soils and exhibits a remarkable tolerance for high salinity, arid conditions, and nutrient-poor substrates, particularly those deficient in nitrogen and phosphorus (Lososová et al., 2009). They can establish themselves in loose substrates containing a small proportion of organic matter, such as sand, ash, and manure (Grüll, 1990). The community transforms on less disturbed sandy substrates into meadow vegetation of the *Festucetea vaginatae* and *Koelerio-Corynephoretea* classes. In ruderal areas, it typically transitions into associations of *Sisymbrion officinalis* and *Atriplicion*, eventually evolving into perennial ruderal vegetation of the *Artemisietea vulgaris* class (Lososová et al., 2009).

Principal component 2 indicates that plants with high trophic resource requirements are more likely to be more hemerobic. The hemeroby index indicates the resilience of communities to the impact of anthropogenic disturbances (Bernhardt-Römermann et al., 2011). The response of vegetation to extreme events depends on the nutrient deficiency tolerance of the species within the community. This tolerance to nutrient deficiencies enhances resilience to extreme disturbances (MacGillivray & Grime, 1995). A higher nutrient content in the soil is likely the result of an increased presence of carbonates (D’Elia et al., 2017), which also contributes to the rise in dissolved salts within the soil. These patterns are of natural origin; however, they create conditions that are conducive to the development of flora resilient to anthropogenic impacts, particularly evident in the context of catastrophic environmental changes. The geological structure of the parent materials of the floodplain soils influences the ratio of sandy deposits, which lack carbonates, to clay deposits that contain higher levels of carbonates (Yakovenko et al., 2023). Soils with varying carbonate contents also exhibit different levels of resistance to water erosion (Berner et al., 2012). This explains why plant associations with low PC2 scores—indicative of high carbonate content—are spatially associated with relatively narrow channels featuring steep clay banks.

The researchers’ attention was drawn to the discovery that in extensive areas of the reservoir where water had receded, there was an almost instantaneous growth of *Salix* × *rubens* and *Populus nigra* communities (Didukh et al., 2024a). The distinctive characteristics of these communities include a relatively higher density and projective cover, a greater degree of naturalness, and a lower level of hemerobic conditions. They also tend to prefer more acidic and well-aerated soils. Furthermore, in comparison to other floodplain habitats, these communities can thrive under conditions of lower soil nutrient content and reduced variability in moisture levels. Didukh et al. (2024a) and Didukh et al. (2024b) observed that the growth rate of *Populus nigra* growing in drier, poorer sandy sediments is significantly lower than that of *Salix* × *rubens*. Our results also indicate that soil nutrient content is the primary factor influencing the differentiation between *Salicetum albae* and *Salici-Populus* associations. Additionally, *Salicetum albae* associations tend to prefer lower soil pH levels compared to those with more frequent *Salici-Populetum* associations. Principal component 3 suggests that increased light conditions favor the development of more hemerobic plants. The *Salicetum albae* and *Salici-Populetum* associations exhibit considerable differences in this component. The *Salicetum albae* association demonstrated a more illuminated environment, which promoted a higher level of hemeroby within this community.

### Conclusion

The destruction of the Kakhovka Dam and the subsequent decrease in water levels in the Dnipro River have led to significant alterations in floodplain ecosystems, particularly on Khortytsia Island. The lowered water levels have created conditions conducive to the formation of new habitats, including sandy open areas and degraded floodplain reservoirs. This disaster has triggered ecological succession processes. Pioneer plant communities are capable of rapidly colonizing the newly exposed areas, while other species are disappearing due to changes in soil moisture, chemical composition, and other environmental factors. The changes in plant cover structure can be analysed through the lens of hemeroby. The sensitivity of plant communities to anthropogenic transformations is influenced by environmental conditions. The drainage of the area has induced changes associated with an increase in the hemeroby of plant communities. Increased light availability and elevated soil nutrient levels resulting from the reservoir drainage are promoting the proliferation of more hemerobic plant species. A substantial part of the drained reservoir area is currently dominated by *Salicetum albae* and *Salici-Populetum* associations. The *Salicetum albae* association exhibits a higher level of hemeroby, likely due to greater light availability compared to the *Salici-Populetum* association. Indicators of hemeroby and naturalness are promising tools for monitoring the dynamics of floodplain ecosystems that have experienced catastrophic anthropogenic changes.

## Supplemental Information

10.7717/peerj.19607/supp-1 Supplemental Information 1 Synoptic phytosociological table. The table shows the percentage of species occurrences within the respective associations. Species for which fidelity was statistically significant for *P*< 0.05 are presented Diagnostic species are marked with an asterisk.
Note: ** I–*Leersio-Bidentetum* (Koch 1926) Poli et Tx. 1960; II–*Chenopodietum rubri* Timár 1950; III–*Cyperetum micheliani* Horvatić 1931; IV–*Phragmitetum australis* Savič 1926; V–*Salicetum albae* Issler 1926; VI–*Salici-Populetum* (Tx. 1931) Meijer-Drees 1936; VII–*Typhetum angustifoliae* Pignatti 1953; VIII–*Portulacetum oleracei* Felföldy 1942; IX–*Xanthietum strumarii* Paucaˇ 1941; X–*Amarantho retroflexi-Echinochloetum cruris-galli* Bagrikova 2005; XI–*Erigeronto-Lactucetum serriolae* Lohmeyer in Oberd. 1957; XII –*Bromo tectorum-Corispermetum leptopteri* Sissingh et Westhoff ex (Sissingh, 1950) corr. Dengler 2000. Other species (numbers of associations where the species was found are in parentheses): *Acer negundo* (I, II, III, IV, VI, VIII), *Aegilops cylindrica* (XI), *Agrostis capillaris* (VI, X), *Ailanthus altissima* (VIII, X, XI), *Althaea officinalis* (II, III, VII, IX), *Ambrosia artemisiifolia* (I, VIII), *Amorpha fruticosa* (I, II, III, IV, V, VI, VII, VIII, IX, X, XI, XII), *Artemisia absinthium* (III, IV, XI), *Artemisia campestris* (X, XI), *Barbarea stricta* (VI), *Bolboschoenus maritimus* (II, III, VIII), *Butomus umbellatus* (I), *Calystegia sepium* (II, III, VI, X), *Cardamine parviflora* (I), *Carduus crispus* (I, VI), *Carex acuta* (I), *Carex hirta* (VI), *Carex spicata* (I, XI), *Celtis occidentalis* (IV, V, VI, IX, X, XI), *Cenchrus longispinus* (VIII, IX, XII), *Centaurea diffusa* (XI), *Chaerophyllum temulum* (VI), *Chondrilla juncea* (V), *Crepis tectorum* (XI), *Cynoglossum officinale* (VI), *Dipsacus fullonum* (VIII), *Eleocharis palustris* (I), *Epilobium parviflorum* (I, IV), *Epilobium tetragonum* (I, VII), *Eragrostis pilosa* (VIII, X), *Fraxinus pennsylvanica* (VI), *Galium aparine* (I), *Geranium palustre* (VI), *Glechoma hederacea* (VI), *Gleditsia triacanthos* (X), *Humulus lupulus* (VI), *Jacobaea erucifolia* (I, XI), *Jacobaea vulgaris* (I, V), *Juncus compressus* (I, II, VIII, X), *Juncus gerardi* (VIII), *Juncus inflexus* (V), *Leonurus quinquelobatus* (VI), *Linaria vulgaris* (VI), *Lipandra polysperma* (II, III, IV), *Lycopus europaeus* (I, II, III, IV, V, VI, VII, IX), *Mentha aquatica* (VI), *Morus alba* (VI), *Myosotis scorpioides* (III), *Myosoton aquaticum* (I, IV), *Oxybaphus nyctagineus* (X, XII), *Persicaria maculosa* (I, II, III, VI, VIII, IX), *Plantago cornuti* (I, IV), *Plantago media* (I), *Poa compressa* (VI, VIII, XI), *Polygonum aviculare* (I, XII), *Robinia pseudoacacia* (IX), *Rorippa amphibia* (VI), *Rumex crispus* (II, VIII, X, XI), *Rumex maritimus* (I, II, IV), *Rumex sylvestris* (III), *Sagittaria sagittifolia* (III), *Salsola tragus* (XI), *Schoenoplectus lacustris* (I, II, VIII), *Scrophularia nodosa* (II, VII), *Secale sylvestre* (VIII, IX, X, XI, XII), *Setaria pumila* (VIII, X), *Setaria viridis* (VIII, X), *Silene latifolia* (XI), *Solanum dulcamara* (II, III, IV), *Solidago canadensis* (IV), *Sonchus arvensis* (I, III, VI, VII), *Sonchus oleraceus* (II), *Sparganium erectum* (II, VII), *Tanacetum vulgare* (I, III, VI), *Tribulus terrestris* (VIII, IX, X, XI), *Tripolium pannonicum* (IV, VII), *Tussilago farfara* (III, IV), *Ulmus minor* (II, VI), *Xanthium albinum* (XII).

10.7717/peerj.19607/supp-2 Supplemental Information 2 Projected cover, species richness, and phytoindication assessments of ecological regimes for various plant associations, along with measures of naturalness and hemeroby (mean ± standard deviation) The letters denote statistically significant differences as determined by the Tukey test for *P* < 0.05. Identical letters indicate the absence of statistically significant differences between associations for the corresponding indicator.
Note: * I–*Leersio-Bidentetum* (Koch 1926) Poli et Tx. 1960; II–*Chenopodietum rubri* Timár 1950; III–*Cyperetum micheliani* Horvatić 1931; IV–*Phragmitetum australis* Savič 1926; V –*Salicetum albae* Issler 1926; VI–*Salici-Populetum* (Tx. 1931) Meijer-Drees 1936; VII–*Typhetum angustifoliae* Pignatti 1953; VIII–*Portulacetum oleracei* Felföldy 1942; IX–*Xanthietum strumarii* Paucaˇ 1941; X–*Amarantho retroflexi-Echinochloetum cruris-galli* Bagrikova 2005; XI–*Erigeronto-Lactucetum serriolae* Lohmeyer in Oberd. 1957; XII–*Bromo tectorum-Corispermetum leptopteri* Sissingh et Westhoff ex (Sissingh, 1950) corr. Dengler 2000.

10.7717/peerj.19607/supp-3 Supplemental Information 3 Matrix with plant species and their projected cover at the recording points

## Additional Information and Declarations

### Data Availability

The following information was supplied regarding data availability: The species matrix is a list of species and their projected coverage at the respective points of record are available in the Supplemental File.
