# Supplementary material for: Hemeroby reveals the dynamics of vegetation cover following the destruction of the Kakhovka Reservoir
Source: PeerJ. 2025 Jun 25;13:e19607. doi: 10.7717/peerj.19607 (PMC12205700; doi:10.7717/peerj.19607)
Supplement: Supplemental Information 1 — Diagnostic species are marked with an asterisk. Note: ** I–Leersio-Bidentetum (Koch 1926) Poli et Tx. 1960; II–Chenopodietum rubri Timár 1950; III–Cyperetum micheliani Horvatić 1931; IV–Phragmitetum australis Savič 1926; V–Salicetum albae Issler 1926; VI–Salici-Populetum (Tx. 1931) Meijer-Drees 1936; VII–Typhetum angustifoliae Pignatti 1953; VIII–Portulacetum oleracei Felföldy 1942; IX–Xanthietum strumarii Paucaˇ 1941; X–Amarantho retroflexi-Echinochloetum cruris-galli Bagrikova 2005; XI–Erigeronto-Lactucetum serriolae Lohmeyer in Oberd. 1957; XII –Bromo tectorum-Corispermetum leptopteri Sissingh et Westhoff ex (Sissingh, 1950) corr. Dengler 2000. Other species (numbers of associations where the species was found are in parentheses): Acer negundo (I, II, III, IV, VI, VIII), Aegilops cylindrica (XI), Agrostis capillaris (VI, X), Ailanthus altissima (VIII, X, XI), Althaea officinalis (II, III, VII, IX), Ambrosia artemisiifolia (I, VIII), Amorpha fruticosa (I, II, III, IV, V, VI, VII, VIII, IX, X, XI, XII), Artemisia absinthium (III, IV, XI), Artemisia campestris (X, XI), Barbarea stricta (VI), Bolboschoenus maritimus (II, III, VIII), Butomus umbellatus (I), Calystegia sepium (II, III, VI, X), Cardamine parviflora (I), Carduus crispus (I, VI), Carex acuta (I), Carex hirta (VI), Carex spicata (I, XI), Celtis occidentalis (IV, V, VI, IX, X, XI), Cenchrus longispinus (VIII, IX, XII), Centaurea diffusa (XI), Chaerophyllum temulum (VI), Chondrilla juncea (V), Crepis tectorum (XI), Cynoglossum officinale (VI), Dipsacus fullonum (VIII), Eleocharis palustris (I), Epilobium parviflorum (I, IV), Epilobium tetragonum (I, VII), Eragrostis pilosa (VIII, X), Fraxinus pennsylvanica (VI), Galium aparine (I), Geranium palustre (VI), Glechoma hederacea (VI), Gleditsia triacanthos (X), Humulus lupulus (VI), Jacobaea erucifolia (I, XI), Jacobaea vulgaris (I, V), Juncus compressus (I, II, VIII, X), Juncus gerardi (VIII), Juncus inflexus (V), Leonurus quinquelobatus (VI), Linaria vulgaris ( [file peerj-13-19607-s001.docx]

| Species | Association** | | | | | | | | | | | | *phi* |
| --- | --- | --- | --- | --- | --- | --- | --- | --- | --- | --- | --- | --- | --- |
|  | I | II | III | IV | V | VI | VII | VIII | IX | X | XI | XII |  |
| *Bidens frondosa* | 100* | 17 | 38 | 83 | 14 | 20 | 25 | 5 | 82 | 31 | – | 14 | 0.40 |
| *Persicaria minor* | 100* | – | – | 33 | 14 | 20 | 12 | – | – | – | – | – | 0.72 |
| *Leersia oryzoides* | 92* | 17 | 62 | 33 | 43 | 27 | 50 | – | – | – | – | – | 0.44 |
| *Persicaria lapathifolia* | 85* | 83 | 25 | 83 | – | 13 | 38 | 29 | 36 | 38 | – | 29 | 0.29 |
| *Carex pseudocyperus* | 69* | 22 | 25 | 50 | 43 | 27 | 12 | – | – | – | – | – | 0.36 |
| *Rumex hydrolapathum* | 69* | 50 | 62 | 33 | – | 33 | 38 | – | – | – | – | – | 0.32 |
| *Oenanthe aquatica* | 54* | – | 12 | – | – | – | – | – | – | – | – | – | 0.64 |
| *Sonchus asper* | 54* | 17 | 25 | – | – | – | – | – | – | – | – | – | 0.51 |
| *Agrostis stolonifera* | 38* | 6 | – | – | 14 | 13 | – | – | – | – | 25 | – | 0.34 |
| *Alopecurus aequalis* | 31* | – | – | – | – | – | – | – | – | – | – | – | 0.54 |
| *Ranunculus repens* | 31* | – | – | – | – | – | – | – | – | – | – | – | 0.54 |
| *Rorippa palustris* | 31* | 6 | – | – | – | – | – | – | – | – | – | – | 0.49 |
| *Oxybasis rubra* | 31 | 100* | 25 | 17 | 14 | – | 12 | 5 | – | – | – | – | 0.67 |
| *Cyperus glomeratus* | 23 | 94* | 38 | – | 14 | – | 38 | 19 | – | – | – | – | 0.58 |
| *Nuphar lutea* | 15 | 72* | 62 | 17 | 29 | 13 | 25 | – | – | – | – | – | 0.40 |
| *Cyperus michelianus* | 31 | 39* | 12 | – | – | – | 12 | – | – | – | – | – | 0.35 |
| *Trapa borysthenica* | – | 33* | – | – | – | 13 | – | 5 | – | – | – | – | 0.43 |
| *Lythrum virgatum* | – | 17* | – | – | – | – | – | – | – | – | – | – | 0.39 |
| *Cyperus fuscus* | 31 | 67 | 100* | – | – | – | 50 | – | – | – | – | – | 0.59 |
| *Iris pseudacorus* | 31 | 6 | 100* | – | – | – | 25 | – | – | – | – | – | 0.77 |
| *Lythrum salicaria* | 15 | 28 | 88* | – | 29 | – | 25 | – | – | – | – | – | 0.60 |
| *Populus alba* | 8 | 22 | 88* | 50 | 29 | – | – | 5 | 9 | – | – | – | 0.56 |
| *Persicaria hydropiper* | – | – | 75* | – | 29 | – | 12 | – | – | – | – | – | 0.67 |
| *Ranunculus sceleratus* | 62 | 6 | 62* | – | – | – | – | – | – | – | – | – | 0.50 |
| *Epilobium roseum* | – | – | 38* | – | 29 | – | – | – | – | – | – | – | 0.42 |
| *Phragmites australis* | 62 | 72 | 75 | 100* | 43 | 73 | 95 | 38 | 27 | 15 | 12 | 43 | 0.27 |
| *Epilobium hirsutum* | 46 | 11 | 50 | 83* | 43 | – | 12 | – | – | – | – | – | 0.47 |
| *Lactuca tatarica* | – | 6 | 50 | 67* | 43 | 33 | – | – | – | – | 12 | – | 0.39 |
| *Carex riparia* | 46 | 11 | 12 | 67* | – | 27 | 50 | – | – | – | – | – | 0.39 |
| *Achillea millefolium* | – | – | 38 | 67* | 57 | 27 | 12 | – | – | – | 25 | – | 0.37 |
| *Elytrigia repens* | – | 6 | – | 50* | 14 | – | – | – | – | – | – | – | 0.57 |
| *Plantago major* | – | 11 | – | 50* | – | 13 | 12 | – | – | – | – | – | 0.50 |
| *Urtica dioica* | – | 6 | – | 33* | 14 | 7 | – | – | – | – | – | – | 0.39 |
| *Cirsium arvense* | 8 | – | 12 | 33* | – | 7 | – | – | – | – | – | – | 0.39 |
| *Carduus acanthoides* | – | – | – | 17* | – | – | – | – | – | – | – | – | 0.39 |
| *Dactylis glomerata* | – | – | – | 17* | – | – | – | – | – | – | – | – | 0.39 |
| *Geum urbanum* | – | – | – | 17* | – | – | – | – | – | – | – | – | 0.39 |
| *Picris hieracioides* | – | – | – | 17* | – | – | – | – | – | – | – | – | 0.39 |
| *Salix rubens* | 69 | 89 | 62 | 17 | 100* | 67 | 62 | 95 | 27 | – | 12 | – | 0.30 |
| *Calamagrostis epigejos* | 8 | – | – | – | 57* | 33 | – | 5 | 9 | – | – | – | 0.50 |
| *Cirsium setosum* | 38 | 11 | 12 | 33 | 57* | 20 | – | – | – | – | – | – | 0.37 |
| *Erigeron annuus* | 31 | – | 38 | 50 | 57* | 13 | 25 | – | – | – | – | – | 0.31 |
| *Cirsium vulgare* | 15 | – | 12 | 33 | 43* | 7 | – | – | – | – | – | – | 0.35 |
| *Poa angustifolia* | – | – | – | – | 29* | – | – | – | – | – | 12 | – | 0.42 |
| *Populus nigra* | 38 | 39 | 50 | 33 | 14 | 100* | 97 | 100 | 82 | 77 | 92 | 86 | 0.20 |
| *Typha angustifolia* | 23 | 11 | 12 | 50 | 57 | 40 | 88* | – | – | – | – | – | 0.46 |
| *Typha latifolia* | 15 | – | 25 | 17 | – | – | 50* | – | – | – | – | – | 0.43 |
| *Portulaca oleracea* | 8 | 22 | 12 | – | – | – | – | 95* | 82 | 69 | – | 43 | 0.46 |
| *Eragrostis minor* | – | – | – | – | – | – | – | 52* | 9 | 8 | – | – | 0.60 |
| *Digitaria sanguinalis* | – | – | – | – | – | – | – | 48* | 45 | 15 | 12 | 14 | 0.35 |
| *Xanthium strumarium* | – | – | – | – | – | – | – | 33 | 100* | 46 | – | 43 | 0.63 |
| *Chenopodium strictum* | 8 | – | – | 17 | – | 7 | – | 57 | 82* | 46 | 38 | – | 0.45 |
| *Amaranthus albus* | – | 17 | – | – | – | – | – | 76 | 82* | 38 | 12 | – | 0.49 |
| *Solanum nigrum* | – | – | 12 | – | – | – | – | – | 55* | 31 | – | – | 0.51 |
| *Chenopodium album* | – | – | 25 | – | – | 7 | – | 10 | 55* | 8 | 12 | – | 0.46 |
| *Convolvulus arvensis* | – | – | – | – | – | – | – | – | 18* | – | – | – | 0.41 |
| *Echinochloa crus–galli* | 46 | 33 | 50 | – | 14 | 7 | – | 33 | 64 | 85* | 12 | 29 | 0.35 |
| *Amaranthus retroflexus* | 15 | 6 | – | – | – | – | – | 67 | 72 | 82* | – | 43 | 0.41 |
| *Erigeron canadensis* | 46 | – | 50 | 83 | 86 | 80 | 38 | 10 | – | 38 | 100* | 29 | 0.32 |
| *Lactuca serriola* | – | – | – | – | 14 | 13 | – | – | – | – | 88* | – | 0.80 |
| *Anisantha sterilis* | – | – | – | – | – | – | – | – | 9 | – | 75* | – | 0.80 |
| *Apera spica–venti* | – | – | 12 | – | 29 | 13 | 12 | 5 | – | – | 62* | – | 0.49 |
| *Corispermum nitidum* | – | – | – | – | – | – | – | 5 | 9 | 8 | 50* | 29 | 0.45 |
| *Oenothera biennis* | – | – | – | – | – | 7 | – | – | – | – | 25* | – | 0.42 |
| *Anisantha tectorum* | – | – | – | – | – | – | – | – | – | – | 38* | 14 | 0.49 |
| *Crepis foetida* | – | – | – | 17 | 14 | 13 | – | – | – | – | 38* | – | 0.37 |
| *Corispermum hyssopifolium* | – | – | – | – | – | – | – | 24 | 18 | 23 | 12 | 100* | 0.72 |
