# Supplementary material for: Hemeroby reveals the dynamics of vegetation cover following the destruction of the Kakhovka Reservoir
Source: PeerJ. 2025 Jun 25;13:e19607. doi: 10.7717/peerj.19607 (PMC12205700; doi:10.7717/peerj.19607)
Supplement: Supplemental Information 2 — The letters denote statistically significant differences as determined by the Tukey test for *P* < 0.05. Identical letters indicate the absence of statistically significant differences between associations for the corresponding indicator. Note: * I–*Leersio-Bidentetum* (Koch 1926) Poli et Tx. 1960; II–*Chenopodietum rubri* Timár 1950; III–*Cyperetum micheliani* Horvatić 1931; IV–*Phragmitetum australis* Savič 1926; V –*Salicetum albae* Issler 1926; VI–*Salici-Populetum* (Tx. 1931) Meijer-Drees 1936; VII–*Typhetum angustifoliae* Pignatti 1953; VIII–*Portulacetum oleracei* Felföldy 1942; IX–*Xanthietum strumarii* Paucaˇ 1941; X–*Amarantho retroflexi-Echinochloetum cruris-galli* Bagrikova 2005; XI–*Erigeronto-Lactucetum serriolae* Lohmeyer in Oberd. 1957; XII–*Bromo tectorum-Corispermetum leptopteri* Sissingh et Westhoff ex (Sissingh, 1950) corr. Dengler 2000. [file peerj-13-19607-s002.docx]

| Variable | Syntaxon* | | | | | | | | | | | |
| --- | --- | --- | --- | --- | --- | --- | --- | --- | --- | --- | --- | --- |
|  | I | II | III | IV | V | VI | VII | VIII | IX | X | XI | XII |
| Cover, % | 56.3 ± 22.7ᵃᵇ | 63.0 ± 17.6ᵃᵇ | 71.6 ± 12.8ᵃ | 65.2 ± 12.0ᵃᵇ | 64.3 ± 23.0ᵃᵇ | 66.4 ± 19.8ᵃ | 77.4 ± 12.4ᵃ | 41.5 ± 11.1ᵇᶜ | 32.4 ± 16.9ᶜᵈ | 23.2 ± 14.8ᶜᵈ | 38.8 ± 11.0ᵇᶜᵈ | 17.6 ± 9.3ᵈ |
| Species | 18.1 ± 5.3ᵃ | 12.2 ± 2.7ᵇᶜ | 18.6 ± 2.7ᵃ | 16.7 ± 2.9ᵃᵇ | 12.7 ± 5.7ᵇᶜ | 10.9 ± 4.1ᶜᵈ | 11.8 ± 3.2ᵇᶜᵈ | 9.7 ± 2.5ᶜᵈ | 11.5 ± 3.3ᵇᶜᵈ | 9.0 ± 2.0ᶜᵈ | 10.9 ± 2.2ᵇᶜᵈ | 6.3 ± 1.1ᵈ |
| Hd, mm | 263.6 ± 29.2ᵃ | 254.3 ± 46.6ᵃ | 254.0 ± 19.4ᵃ | 202.9 ± 34.3ᵃᵇ | 174.0 ± 52.9ᵇ | 179.2 ± 59.0ᵇ | 264.2 ± 38.4ᵃ | 107.2 ± 23.3ᶜ | 111.4 ± 18.9ᶜ | 99.1 ± 19.0ᶜ | 75.8 ± 7.8ᶜ | 85.8 ± 15.1ᶜ |
| fH, dimensionless unit | 0.3 ± 0.1ᵇᶜ | 0.3 ± 0.1ᵇ | 0.3 ± 0.1ᵇᶜ | 0.3 ± 0.1ᵇᶜ | 0.3 ± 0.1ᵇᶜ | 0.3 ± 0.1ᵇᶜ | 0.2 ± 0.1ᶜ | 0.4 ± 0.1ᵃ | 0.4 ± 0.1ᵃ | 0.4 ± 0.1ᵃ | 0.4 ± 0.1ᵃ | 0.4 ± 0.1ᵃ |
| Rc, pH | 6.3 ± 0.2ᶜ | 6.4 ± 0.2ᵇᶜ | 6.4 ± 0.2ᵃᵇᶜ | 6.6 ± 0.1ᵃᵇ | 6.3 ± 0.2ᶜ | 6.5 ± 0.2ᵃᵇ | 6.5 ± 0.2ᵃᵇ | 6.3 ± 0.2ᵇᶜ | 6.4 ± 0.1ᵃᵇᶜ | 6.4 ± 0.3ᵃᵇᶜ | 6.7 ± 0.1ᵃ | 6.5 ± 0.1ᵃᵇᶜ |
| Sl, μg/l | 21.5 ± 4.2ᵇᶜ | 24.7 ± 6.2ᵃᵇᶜ | 24.1 ± 5.8ᵃᵇᶜ | 25.5 ± 4.9ᵃᵇᶜ | 21.5 ± 3.4ᵇᶜ | 21.2 ± 3.0ᵇᶜ | 23.1 ± 5.7ᵃᵇᶜ | 18.1 ± 5.1ᶜ | 24.5 ± 8.0ᵃᵇᶜ | 27.6 ± 8.8ᵃᵇ | 31.4 ± 10.2ᵃ | 25.9 ± 11.2ᵃᵇᶜ |
| Ca, CaO+MgO % | 0.4 ± 0.1ᶜᵈ | 0.3 ± 0.2ᵈ | 0.3 ± 0.1ᶜᵈ | 1.1 ± 0.7ᵃ | 0.7 ± 0.5ᵃᵇᶜ | 0.6 ± 0.3ᵇᶜᵈ | 0.3 ± 0.2ᶜᵈ | 0.4 ± 0.2ᶜᵈ | 0.6 ± 0.3ᵇᶜᵈ | 0.7 ± 0.3ᵃᵇᶜ | 0.8 ± 0.4ᵃᵇ | 0.4 ± 0.2ᶜᵈ |
| Nt, g/kg | 3.9 ± 0.2ᵃ | 3.4 ± 0.5ᵃᵇ | 3.9 ± 0.2ᵃ | 3.6 ± 0.3ᵃᵇ | 3.8 ± 1.0ᵃᵇ | 2.6 ± 0.8ᶜᵈᵉ | 3.0 ± 0.6ᵃᵇᶜᵈ | 2.5 ± 0.9ᶜᵈᵉ | 3.4 ± 0.6ᵃᵇ | 3.1 ± 0.6ᵃᵇᶜ | 1.8 ± 0.5ᵉ | 1.9 ± 1.3ᵈᵉ |
| Ae, % | 14.8 ± 3.9ᵉ | 13.3 ± 3.3ᵉ | 18.1 ± 4.2ᵈᵉ | 21.2 ± 6.7ᵈᵉ | 39.0 ± 16.5ᵇᶜᵈ | 36.0 ± 19.3ᶜᵈ | 13.7 ± 6.3ᵉ | 54.0 ± 18.0ᵇ | 51.8 ± 22.8ᵇᶜ | 48.9 ± 17.6ᵇᶜ | 76.6 ± 6.1ᵃ | 53.7 ± 19.6ᵇᶜ |
| Tm, *gJ* m–^2^ year^–1^ | 2.0 ± 0.1ᵇᶜ | 1.9 ± 0.1ᶜ | 1.9 ± 0.1ᶜ | 2.3 ± 0.1ᵃᵇ | 2.1 ± 0.2ᵃᵇᶜ | 2.2 ± 0.2ᵃᵇ | 2.2 ± 0.3ᵃᵇᶜ | 2.2 ± 0.2ᵃᵇ | 2.3 ± 0.1ᵃ | 2.2 ± 0.2ᵃᵇ | 2.1 ± 0.1ᵃᵇᶜ | 1.9 ± 0.1ᶜ |
| Om, mm | –0.1 ± 0.2ᵃ | –1.0 ± 0.4ᵇᶜ | –0.5 ± 0.2ᵃᵇ | –0.6 ± 0.6ᵃᵇ | –1.1 ± 0.7ᵇᶜᵈ | –1.0 ± 0.8ᵇᶜ | –0.9 ± 0.3ᵃᵇᶜ | –2.5 ± 0.8ᵉ | –1.4 ± 0.4ᵇᶜᵈ | –2.0 ± 1.0ᵈᵉ | –1.8 ± 0.8ᶜᵈᵉ | –1.6 ± 0.4ᵇᶜᵈ |
| Kn, dimensionless unit | 106.8 ± 3.4ᵈ | 110.7 ± 16.6ᶜᵈ | 100.1 ± 5.5ᵈ | 117.9 ± 23.7ᵇᶜᵈ | 112.4 ± 26.8ᵇᶜᵈ | 110.1 ± 14.0ᶜᵈ | 104.1 ± 6.8ᵈ | 130.3 ± 26.7ᵇᶜᵈ | 134.6 ± 14.6ᵃᵇᶜ | 138.8 ± 22.5ᵃᵇ | 132.4 ± 19.1ᵃᵇᶜᵈ | 164.1 ± 35.8ᵃ |
| Cr, °С | 0.4 ± 1.2ᵃᵇᶜ | –3.7 ± 1.3ᵈᵉ | –2.2 ± 1.8ᵇᶜᵈᵉ | –2.7 ± 4.2ᵇᶜᵈᵉ | –2.0 ± 2.4ᵇᶜᵈᵉ | –3.0 ± 2.8ᶜᵈᵉ | –1.8 ± 2.1ᵇᶜᵈᵉ | 1.8 ± 5.0ᵃᵇ | 3.8 ± 3.0ᵃ | –0.3 ± 3.6ᵃᵇᶜᵈ | 0.3 ± 2.0ᵃᵇᶜᵈ | –7.0 ± 4.0ᵉ |
| Lc, % | 81.1 ± 4.4ᵇ | 90.9 ± 4.3ᵃ | 84.5 ± 6.3ᵃᵇ | 86.2 ± 5.4ᵃᵇ | 90.1 ± 3.6ᵃ | 85.8 ± 8.7ᵃᵇ | 89.0 ± 7.0ᵃ | 93.1 ± 3.3ᵃ | 89.0 ± 3.1ᵃ | 91.7 ± 5.1ᵃ | 90.9 ± 5.2ᵃ | 93.3 ± 4.1ᵃ |
| Light Regime | 7.1 ± 0.2ᵇᶜ | 7.6 ± 0.4ᵃ | 7.3 ± 0.2ᵃᵇ | 7.2 ± 0.3ᵃᵇ | 7.7 ± 0.2ᵃ | 6.8 ± 0.4ᵇᶜ | 7.1 ± 0.4ᵃᵇᶜ | 7.0 ± 0.4ᵇᶜ | 7.1 ± 0.4ᵇᶜ | 7.0 ± 0.5ᵇᶜ | 6.5 ± 0.3ᶜ | 7.7 ± 0.3ᵃ |
| Temperatures | 4.6 ± 0.1ᶜᵈᵉ | 4.5 ± 0.2ᵉ | 4.6 ± 0.1ᶜᵈᵉ | 4.7 ± 0.1ᵇᶜᵈᵉ | 4.5 ± 0.1ᵈᵉ | 4.7 ± 0.2ᵇᶜᵈ | 4.7 ± 0.1ᵇᶜᵈᵉ | 4.9 ± 0.2ᵇ | 5.2 ± 0.1ᵃ | 5.2 ± 0.2ᵃ | 5.0 ± 0.1ᵃᵇ | 4.9 ± 0.2ᵇᶜ |
| Continentality of Climate | 7.8 ± 0.3ᵇᶜ | 7.5 ± 1.4ᶜ | 7.9 ± 0.5ᵇᶜ | 8.8 ± 0.2ᵃᵇ | 8.2 ± 0.6ᵃᵇᶜ | 7.5 ± 0.8ᶜ | 8.1 ± 0.4ᵃᵇᶜ | 8.0 ± 0.7ᵇᶜ | 9.1 ± 0.8ᵃ | 9.1 ± 0.9ᵃ | 8.1 ± 0.3ᵃᵇᶜ | 9.0 ± 0.7ᵃᵇ |
| Humidity | 6.4 ± 0.3ᵃᵇ | 6.9 ± 0.6ᵃ | 6.6 ± 0.4ᵃᵇ | 5.6 ± 0.6ᶜᵈᵉ | 5.4 ± 0.6ᶜᵈᵉ | 6.1 ± 0.7ᵇᶜ | 6.9 ± 0.5ᵃᵇ | 5.6 ± 0.6ᶜᵈ | 4.8 ± 0.5ᵉᶠ | 4.7 ± 0.7ᵉᶠ | 5.3 ± 0.2ᵈᵉ | 4.3 ± 0.7ᶠ |
| Acidity | 6.3 ± 0.1ᵈᵉ | 6.5 ± 0.1ᵇᶜ | 6.5 ± 0.1ᶜᵈᵉ | 6.5 ± 0.2ᵇᶜᵈ | 6.2 ± 0.1ᵉ | 6.7 ± 0.2ᵃᵇᶜ | 6.6 ± 0.2ᵃᵇᶜ | 6.7 ± 0.2ᵃ | 6.7 ± 0.1ᵃᵇᶜ | 6.5 ± 0.2ᵇᶜ | 6.9 ± 0.1ᵃ | 6.7 ± 0.1ᵃᵇ |
| Nutrients Availability | 6.9 ± 0.2ᵃ | 5.7 ± 0.8ᵇᶜ | 6.4 ± 0.4ᵃᵇᶜ | 6.5 ± 0.4ᵃᵇᶜ | 5.6 ± 0.4ᶜ | 6.2 ± 0.6ᵃᵇᶜ | 6.6 ± 0.5ᵃᵇ | 5.8 ± 0.5ᵇᶜ | 6.5 ± 0.5ᵃᵇᶜ | 6.3 ± 0.6ᵃᵇᶜ | 6.5 ± 0.3ᵃᵇᶜ | 4.5 ± 1.1ᵈ |
| Naturalness | 1.2 ± 0.6ᵇᶜᵈ | 3.7 ± 1.0ᵃ | 1.7 ± 1.0ᵇᶜ | 0.2 ± 1.4ᶜᵈᵉ | 0.1 ± 1.2ᶜᵈᵉ | 2.4 ± 1.8ᵃᵇ | 1.8 ± 1.1ᵇᶜ | 2.0 ± 1.2ᵇ | –0.2 ± 1.2ᵈᵉ | –0.5 ± 1.0ᵉ | 2.8 ± 0.5ᵃᵇ | –0.7 ± 0.8ᵉ |
| Hemeroby | 48.4 ± 3.9ᵇᶜ | 38.2 ± 8.5ᶜ | 42.0 ± 3.9ᶜ | 61.8 ± 12.1ᵃᵇ | 64.8 ± 11.7ᵃ | 44.4 ± 16.2ᶜ | 39.3 ± 9.9ᶜ | 48.5 ± 11.5ᵇᶜ | 64.8 ± 10.8ᵃ | 65.8 ± 11.0ᵃ | 42.2 ± 4.4ᶜ | 63.0 ± 6.5ᵃᵇ |
